# Is Aberrant Reno-Renal Reflex Control of Blood Pressure a Contributor to Chronic Intermittent Hypoxia-Induced Hypertension?

**DOI:** 10.3389/fphys.2019.00465

**Published:** 2019-04-24

**Authors:** Sara AlMarabeh, Mohammed H. Abdulla, Ken D. O'Halloran

**Affiliations:** Department of Physiology, School of Medicine, College of Medicine and Health, University College Cork, Cork, Ireland

**Keywords:** obstructive sleep apnoea syndrome, intermittent hypoxia, reno-renal reflexes, renal afferent nerves, neurogenic hypertension, sympathoexcitation

## Abstract

Renal sensory nerves are important in the regulation of body fluid and electrolyte homeostasis, and blood pressure. Activation of renal mechanoreceptor afferents triggers a negative feedback reno-renal reflex that leads to the inhibition of sympathetic nervous outflow. Conversely, activation of renal chemoreceptor afferents elicits reflex sympathoexcitation. Dysregulation of reno-renal reflexes by suppression of the inhibitory reflex and/or activation of the excitatory reflex impairs blood pressure control, predisposing to hypertension. Obstructive sleep apnoea syndrome (OSAS) is causally related to hypertension. Renal denervation in patients with OSAS or in experimental models of chronic intermittent hypoxia (CIH), a cardinal feature of OSAS due to recurrent apnoeas (pauses in breathing), results in a decrease in circulating norepinephrine levels and attenuation of hypertension. The mechanism of the beneficial effect of renal denervation on blood pressure control in models of CIH and OSAS is not fully understood, since renal denervation interrupts renal afferent signaling to the brain and sympathetic efferent signals to the kidneys. Herein, we consider the currently proposed mechanisms involved in the development of hypertension in CIH disease models with a focus on oxidative and inflammatory mediators in the kidneys and their potential influence on renal afferent control of blood pressure, with wider consideration of the evidence available from a variety of hypertension models. We draw focus to the potential contribution of aberrant renal afferent signaling in the development, maintenance and progression of high blood pressure, which may have relevance to CIH-induced hypertension.

## Introduction

Sleep apnoea patients experience periodic interruption of ventilation during sleep epochs accompanied by arterial hypoxaemia, hypercapnia, and sleep fragmentation. Recurrent apnoeas can arise from occlusions of the upper airway giving rise to obstructive sleep apnoea syndrome (OSAS) or periodic suppression of central respiratory drive causing central sleep apnoea (Prabhakar and Kumar, 2010; Javaheri and Dempsey, 2013). During apnoea, hypoxaemia stimulates arterial chemoreceptors in the carotid bodies which transduce afferent signals to hindbrain regions enhancing sympathetic discharge to peripheral organs (Loredo et al., 2001). Reflex sympathoexcitation increases cardiac output, which facilitates oxygen supply to essential organs. OSAS patients experience nocturnal hypertension (Kario et al., 2016b). Hypertension accompanied by elevated sympathetic nervous activity persists even during normal oxygenation giving rise to diurnal hypertension (Nagata et al., 2008; Witkowski et al., 2011; Sharpe et al., 2013). Indeed, OSAS is an independent risk factor for hypertension and is associated with high incidence of cardiovascular co-morbidities such as ischaemic heart disease, heart failure, arrhythmia, and angina (Gottlieb et al., 2010; Shah et al., 2010; Konecny et al., 2014). Clinical trials addressing the relationship between OSAS and hypertension estimate that 30–70% of OSAS patients are hypertensive (Ahmad et al., 2017). The combined evidence points to a strong causal relationship between OSAS and hypertension.

Exposure to recurrent intermittent hypoxia (IH) of varying severity and duration is considered the primary stimulus causing autonomic dysregulation in OSAS patients. Experimental animal models of IH consistently demonstrate persistent increases in arterial blood pressure (Fletcher et al., 1992; Lin et al., 2007; Zoccal et al., 2007b; Sharpe et al., 2013; Yamamoto et al., 2013; Lucking et al., 2014; Peng et al., 2014; Del Rio et al., 2016; O'Neill et al., 2019). Indeed, animal models of chronic IH (CIH) are widely used in the study of pathophysiological processes associated with sleep apnoea. Exposure to IH increases sympathetic outflow as evidenced by observations of IH-induced increases in muscle, renal, and splanchnic sympathetic nerve activity and increased vascular tone (Narkiewicz et al., 1998; Soukhova-O'Hare et al., 2006; Peng and Prabhakar, 2008; Silva and Schreihofer, 2011). Ganglion blockade results in a greater reduction of blood pressure in CIH-exposed animals compared with controls, which highlights that sympathoexcitaton is the primary cause of high blood pressure in this model (Zoccal et al., 2009a). Table 1 summarizes the details of a variety of CIH protocols utilizing different hypoxic durations and intensities that evoke cardiorespiratory changes.

**Table 1:** Changes in blood pressure and sympathetic nerve activity during exposure to different protocols of chronic intermittent hypoxia (CIH).

**Species**	**Duration of exposure to IH (days, h/day)**	**Frequency of exposure (cycles/h)**	**Intensity of hypoxia (% of O_**2**_)**	**Blood pressure increase: CIH vs. control (%)**	**Sympathetic nerve activity**	**Catecholamine levels: CIH vs. control**	**References**
Human	28, 9 h/day	~17	13%	4.2%^b^	↑ MSNA	–	Gilmartin et al., 2010
Human	14, 8 h/day	30	First day, 15%. Remaining 13 days, 13%	6.3%^a^ 7.1%^b^ 7.9%^c^	↑ MSNA	–	Tamisier et al., 2011
Rats	35, 6–8 h/day	60	2–3%	12%^c^	Decreased vascular responsiveness to Ach, but no change in vascular response to NE	–	Tahawi et al., 2001
Rats	35, 8 h/day	10	10%	16%^a^ 27.2%^b^ 22.5%^c^	No change in aortic responses to Ach or phenylephrine	–	Ribon-Demars et al., 2018
Rats	35, 8 h/day	6.6	6%	9.1%^a^ 9.3%^b^ 8.0%^c^	An increase in vascular sympathetic activity	↑ 53.2% in NE levels, no change in epinephrine levels.	Zoccal et al., 2007b
Rats	30, 12 h/day	20	8%	No change	–	No change	Soukhova-O'Hare et al., 2006
Rats	30, 6 h/day	48	2–5%	17.5^c^ 18.9^c^	–	–	Lai et al., 2006
Rats	28, 12 h/day	15	10%	17.3%^c^	↑ RSNA	–	Marcus et al., 2010
Rats	28, 8 h/day	40	5%	20.5%^a^	↑ RSNA	–	Lu et al., 2017b
Rats	21, 8 h/day	15	8–10%	No change	–	–	Huang et al., 2010
Rats	21, 8 h/day	40	5%	16%^a^	↑ RSNA	↑ serum NE	Lu et al., 2017a
Rats	21, 8 h/day	30	9%	18.5%^a^	–	–	Guo et al., 2013
Rats	21, 8 h/day	12	5%	24.4%^a^ 9.0%^b^	–	–	Del Rio et al., 2012
Rats	14, 12 h/day	15	10%	6.7%^c^	- Decreased myogenic contractility of arteries - Decreased vascular responsiveness to NE, but not Ang II	–	Phillips et al., 2006
Rats	14, 8 h/day	12	5%	9.2%^a^	No change in vascular conductance in response to lumbar sympathetic stimulation		Lucking et al., 2014
Rats	14, 8 h/day	6.6	6%	7.4%^c^	↑ Splanchnic SNA	–	Silva and Schreihofer, 2011
Rats	14, 7 h/day	20	5%	14.4%^c^	–	–	Troncoso Brindeiro et al., 2007
Rats	10, 8 h/day	6.6	6%	7.0%^a^ 10%^b^ 11.9%^c^	↑ tSNA during late expiration	–	Zoccal et al., 2008
Rats	10, 8 h/day	6.6	6%	12.2%^a^ 11.8%^c^	An increase in vascular sympathetic activity	–	Zoccal et al., 2009a
Rats	7, 8 h/day	10	10%	10.9%^c^	↑ LSNA	–	Sharpe et al., 2013
Rats	7, 8 h/day	6	9%	8.7%^c^	–	–	Yamamoto et al., 2013
Rats	6, 6 h/day	48	2–5%	17.5^c^ 18.9^c^	–	–	Lai et al., 2006
Mice	180, 12 h/day	10	5.7%	20.1%^c^	–	–	Lin et al., 2007
Mice	35, 8 h/day	20	10%	8.7%^a^ 11.3%^c^		–	Coleman et al., 2010
Mice	28, 8 h/day	12	10%	–	–	↑ urine NE	Keiko et al., 2017
Mice	14, 8 h/day	20	10%	No change	–	–	Coleman et al., 2010

*RSNA, renal sympathetic nerve activity; LSNA, lumbar sympathetic nerve activity; MSNA, muscle sympathetic nerve activity; SpSNA, splanchnic sympathetic nerve activity*.

a Systolic blood pressure,

b Diastolic blood pressure,

c *Mean blood pressure; ↑, an increase in corresponding SNA or catecholamines*.

Under physiological conditions, acute manipulations of blood pressure stimulate high- and low-pressure baroreceptors (Kawada et al., 2011; Oga et al., 2018). Sensory inputs are integrated in the nucleus tractus solitarius (NTS) in the dorsal medulla of the brainstem, with resultant inhibition of sympathetic nervous outflow and activation of parasympathetic outflow causing blood pressure to be restored to normal (Accorsi-Mendonça and Machado, 2013). Within minutes to hours, arterial blood pressure elevation is followed by a significant increase in urinary output of sodium and water, that is pressure natriuresis and pressure diuresis, respectively (Hall, 1994). Moreover, there are accompanying changes in the hormonal and neural regulation of the kidneys affecting the control of body fluids, highlighting the role of the kidneys as major determinants of long-term body fluid homeostasis. Electrolyte imbalance inevitably occurs if there is a loss of kidney function with deleterious consequences for blood pressure control. In OSAS, a variety of mechanisms such as endothelial dysfunction, inflammation, atherosclerosis, and fibrosis are suggested to adversely affect renal function resulting in kidney damage (Adeseun and Rosas, 2010). Of note, OSAS is present in 50-70% of patients with end-stage renal disease (ESRD) (Ozkok et al., 2014). Patients with OSAS and concomitant chronic kidney disease or ESRD are significantly more likely to develop resistant hypertension (Abdel-Kader et al., 2012). The interplay between the kidneys and hypertension in OSAS was demonstrated in a study by Nicholl et al. (2014) showing that treatment of OSAS patients with continuous positive airway pressure (CPAP), which resolves recurrent apnoeas, results in a reduction in renin-angiotensin-aldosterone system (RAAS) activity. Importantly, the kidneys are richly innervated by afferent and efferent sympathetic nerves, which play a key role in the maintenance of normal water and electrolyte balance and renin release in a mechanism known as the reno-renal reflex (Johns et al., 2011). Inappropriate afferent signaling through renal afferent nerves is suggested to interfere with the normal regulation of sympathetic outflow and exacerbation of efferent nerve activity leading to hypertension in animal models of renal injury and inflammation (Abdulla and Johns, 2017). This concept is further supported in a recent review (Patinha et al., 2017). Subsequently, enhanced efferent nerve activity arising from RAAS activation leads to endothelial dysfunction and pathophysiological renal changes associated with chronic hypertension and chronic kidney disease (Aroor et al., 2013). Angiotensin II (Ang II) causes renal vasoconstriction, decreasing renal blood flow, lowering renal cortical oxygen tension (PO_2_), which causes renal oxidative stress (Welch et al., 2005; Emans et al., 2016). Ang II actions are mediated by enhanced activity of NADPH oxidase (NOX) and decreased superoxide dismutase (SOD) activity (Welch et al., 2005). This leads to increased levels of reactive oxygen species (ROS), which causes the activation of inflammasomes that cause tubulointerstitial fibrosis, in addition to a decrease in nitric oxide (NO) bioavailability and uncoupling of endothelial nitric oxide synthase (eNOS) as seen in CIH models (Badran et al., 2016; Sogawa et al., 2018). An increase in renal oxidative stress was reported in CIH-exposed animals, an outcome which precedes kidney injury (Lu et al., 2017b; Poonit et al., 2017). Similarly, a recent study in rats demonstrated that exposure to CIH provokes kidney injury by an oxidative stress-related mechanism that involves an imbalance in hypoxia-dependent transcriptional regulation and downregulation of antioxidant defense (Poonit et al., 2017). The resultant oxidative stress enhances hypoxia inducible factor-1 (HIF-1) and the expression of genes including tumor necrosis factor (TNF-α) and erythropoietin, which are implicated in kidney fibrosis. Exposure to IH in a mouse model was associated with renal structural changes such as glomerular hypertrophy, mesangial matrix expansion, increased expression of glomerular growth factors and increased cellular apoptosis (Abuyassin et al., 2018).

In patients with OSAS and attendant hypertension, catheter-based renal denervation results in a significant decrease in blood pressure (Witkowski et al., 2011; Kario et al., 2016a). In 15 patients with moderate-to-severe OSAS, Zhao et al. (2013) reported a mean reduction of about 12 and 5 mmHg in daytime systolic and diastolic blood pressure, respectively, 30 days after catheter-based renal denervation. In the same study, 16 other patients were treated with continuous positive airway pressure, which lowered systolic and diastolic blood pressure after 30 days of treatment. Both catheter-based renal denervation and CPAP resulted in an improvement in apnoea-hypopnea index (AHI) and increased oxygen saturation. CPAP treatment was more efficacious than renal denervation in lowering AHI (approximately 30 events/hr vs. 5 events/hr) and improving oxygen saturation. However, renal denervation was more efficacious than CPAP in lowering systolic and diastolic blood pressure (Zhao et al., 2013). Similarly, a study by Witkowski et al. (2011) showed a median reduction of ~34 and ~13 mmHg in daytime systolic and diastolic blood pressure, respectively, 6 months following catheter-based renal denervation in 10 patients with mild (AHI <15/hour) and moderate-to-severe OSAS (AHI>15/hour). The study also reported an improvement in AHI up to 6 months after renal denervation. However, half of the patients included in this study had mild OSAS (AHI <,15/hour) and two participants received concomitant CPAP therapy. Daniels et al. (2017) conducted a renal denervation study on 20 patients with moderate-to-severe-OSAS, not receiving CPAP treatment. No significant improvement in AHI or oxygen saturation was observed 6 months following catheter-based denervation. Noteworthy, however, was the observation of a significant decrease in systolic and diastolic blood pressure (office and 24 h-ambulatory measurements) (Daniels et al., 2017).

Kario et al. (2016a) carried out a large, blinded and randomized study on patients with resistant hypertension (systolic blood pressure ≥ 160 mmHg, administered 3 or more anti-hypertensive medications including a diuretic, at maximum doses). In this Simplicity HTN-3 trial, 364 patients (26%, 91 patients with OSAS) had catheter-based renal denervation surgery and 171 patients (32%, 54 patients with OSAS) were controls. Office and 24 h-ambulatory blood pressure were measured 6 months after the denervation surgery. OSAS-resistant hypertensive patients showed a significant reduction in office, but not ambulatory systolic blood pressure 6 months after renal denervation. In addition, a significant decrease in the maximum night-time systolic blood pressure was observed. However, patients included in this study had self-reported OSAS. Recently, Warchol-Celinska et al. (2018) conducted a randomized study on 60 patients diagnosed by polysmnography with moderate-to-severe OSAS and resistant hypertension. Half of the participants underwent a catheter-based renal denervation procedure. Three months following denervation, a significant decrease in office systolic (−22 mmHg) and diastolic (−8 mmHg) blood pressure was reported. Moreover, a significant −12 mmHg decrease in 24 h-ambulatory systolic blood pressure and −7 mmHg decrease in 24 h-ambulatory diastolic blood pressure was observed. A decrease in night-time systolic blood pressure and heart rate was also reported. An improvement in AHI was shown in patients with and without concomitant CPAP therapy (Warchol-Celinska et al., 2018). The mechanism by which renal denervation results in improvement of OSAS severity is not clearly understood, but it might be related to a decrease in sodium and water retention; thus, less rostral fluid shift during sleep in the supine posture. In addition, renal denervation interrupts the afferent signals traveling centrally from the kidney. The improvement might be also related indirectly to blood pressure lowering effects, which attenuate chemoreflexes that modulate breathing.

A systematic review was reported in 2015 that included 5 studies of the effects of renal denervation on OSAS-hypertensive patients. The meta-analysis reported 49 patients that showed a significant improvement in AHI 6 months after catheter-based renal denervation. Three out of the five studies reported a significant lowering of office systolic blood pressure (~-15 mmHg) 6 months after renal denervation (Shantha and Pancholy, 2015). It is suggested that failure in nocturnal blood pressure dipping in sleep apnoea is a risk factor for cardiovascular outcomes; thus, this index is a more potent predictor of cardiovascular events than daytime blood pressure (Calhoun, 2010). A recent meta-analysis of 10 randomized clinical trials conducted between 1946 and 2017 with a total of 7,266 participants has been published (Yu et al., 2017). It showed no association between cardiovascular events (stroke, acute coronary syndrome and unstable angina) or number of deaths with the use of CPAP therapy (Yu et al., 2017). It appears that CPAP is not a preventive treatment for serious cardiovascular outcomes that are associated with sleep apnoea. On the other hand, it was shown that OSAS is positively correlated with elevated plasma aldosterone levels (Pratt-Ubunama et al., 2007). Aldosterone induces fluid retention, which can cause intra-vascular fluid expansion increasing upper airway resistance. The addition of spironolactone to the normal anti-hypertensive regimen in 12 moderate-to-severe OSAS patients with resistant hypertension was associated with a 50% decrease in AHI. It is suggested that OSAS contributes to aldosterone secretion, which exacerbates hypertension in the advanced stages of sleep apnoea (Calhoun, 2010).

During apnoea, hypoxia and hypercapnia stimulate chemoreflexes, which increase sympathetic drive causing blood pressure elevation. Renal denervation eliminates the increase in renal sympathetic over-activity, which otherwise causes chronic fluid retention and blood pressure elevation. However, the mechanisms that result in suppression of high blood pressure development and progression following denervation are unclear and many questions remain. This review discusses the link between CIH-induced renal injury and hypertension, with a focus on putative changes in reno-renal reflex mechanisms controlling arterial blood pressure. We summarize the current understanding of the mechanisms involved in CIH-induced hypertension as a model of OSAS. In addition, we draw focus to kidney injury and reno-renal reflexes that can contribute to neurogenic hypertension, with potential relevance to CIH-induced hypertension, and hence OSAS.

## Mechanisms of CIH-Induced Hypertension

CIH protocols that vary in episode frequency, intensity of hypoxia, and duration are associated with different cardiorespiratory outcomes. Duration of exposure to CIH has a major impact on blood pressure, which correlates with elevated sympathetic activity and increased levels of catecholamines (Table 1). For a given duration, increased hypoxia intensity or increased number of hypoxia cycles also correlate with increased blood pressure. Whereas, exposure to CIH typically produces a hypertensive phenotype, different protocols may evoke different mechanisms associated with the development and maintenance of hypertension. A review of the current proposed mechanisms based upon experimental evidence is provided below.

### Vascular Dysfunction and Cardiac Output

Increased cardiac output and peripheral arterial resistance result in blood pressure elevation. Both factors are influenced by changes in sympathetic nervous activity. In CIH, elevated sympathetic nerve activity facilitates catecholamine release from the adrenal medullae, which causes peripheral vasoconstriction stimulating renin release (Zoccal et al., 2007b; Kumar et al., 2015). Endothelin (ET) is reported to be increased in experimental models of CIH and in patients with OSAS. ET elicits vasoconstriction when it activates ET-A receptors and vasodilation through its action on endothelial ET-B receptors (Schneider et al., 2007). In an *in vitro* study using human vascular endothelial cells, hypoxia was shown to enhance ET gene expression leading to increased secretion of ET (Lanfranchi and Somers, 2001). Immunohistochemistry and western blotting studies in rats revealed an upregulation of ET-A receptors in the rat aorta after 3 weeks of exposure to CIH (Guo et al., 2013). In addition, there was a downregulation of ET-B receptors, which are known to mediate vasodilation through a mechanism involving NO. A reduction in NO bioavailability and downregulation of neuronal nitric oxide synthase (nNOS) protein expression in CIH-exposed rats was reported by Marcus et al. (2010). Greater vasoconstriction was attained when an NOS inhibitor was applied to sham animals, which indicates low basal NO bioavailability in CIH-exposed animals (Tahawi et al., 2001). *In vivo* studies reported an overexpression of ET-A receptors in the subfornical organs (SFO) of CIH-exposed animals with an associated increase in blood pressure by 40% compared with 9% in the sham animals when intracerebroventricular ET-1 was administered (Huang et al., 2010). ET-A receptor-dependent hypertension was found to be related to oxidative stress, since pretreatment with a SOD mimetic, tempol, attenuated the elevation of blood pressure in CIH-exposed rats (Troncoso Brindeiro et al., 2007). Interestingly, a similar increase in ET-1 and ET receptor expression was observed in patients with OSAS (Gjørup et al., 2007, 2008). However, treatment with an antioxidant, carbocysteine, improved AHI and respiratory parameters in OSAS patients, but did not affect ET-1 levels (Wu K. et al., 2016).

Exposure to CIH for 35 days resulted in a significant increase in plasma corticosterone, which can enhance the vasoconstrictor response to ET-I, Ang II and catecholamines (Zoccal et al., 2007a). However, ET-1 and norepinephrine (NE) application on cremaster muscle evoked similar vasoconstrictor responses after 35 days of exposure to CIH compared with sham rats (Tahawi et al., 2001). In contrast, responsiveness of gracilis arterioles to NE was significantly less in CIH-exposed rats, which may be due to elevated levels of superoxide in CIH-exposed animals as tempol restored the vasoconstrictor response to NE (Phillips et al., 2006). An increase in gracilis arteriolar stiffness was reported, which was also eliminated by tempol treatment (Phillips et al., 2006). Impaired vasodilatory response of gracilis arteries and cremaster muscle arteries to acetylcholine was reported in CIH-exposed rats (Tahawi et al., 2001; Marcus et al., 2012). Treatment with losartan restored the normal responsiveness to acetylcholine suggesting a role for Ang II in impaired vascular reactivity of CIH-exposed rats. In addition, AT1:AT2 receptor expression was elevated in CIH-exposed rats compared with sham rats (Marcus et al., 2012). Administration of N-acetylcysteine relieved blood pressure elevation in CIH-exposed rats. Increased KCl-mediated constriction of femoral arteries in CIH-exposed rats was partially reduced following N-acetylcysteine treatment and completely reversed following combination treatment of N-acetylcysteine and an arginase inhibitor. The same combination treatment was associated with a complete restoration of NOS-dependent relaxation of femoral and carotid arteries in response to acetylcholine (Krause et al., 2018). This suggests that N-acetylcysteine works on mechanisms other than vascular endothelial function. Moreover, the findings suggest that impaired endothelial expression of eNOS and arginase 1 is partly responsible for endothelial dysfunction in CIH-exposed rats. Therefore, development of CIH-induced hypertension appears partly related to an oxidative stress mechanism with resultant vascular dysfunction. On the other hand, 35 days of exposure to CIH did not alter oxidative and anti-oxidative enzymes activities in the aorta of rats. Aortic ring responsiveness to acetylcholine and phenylephrine was not altered after exposure to CIH with no increase in ET-1 levels in the systemic circulation (Ribon-Demars et al., 2018), although these divergent findings may relate to differences between conduit and resistance vessels. Mechanisms of endothelial dysfunction in CIH models and OSAS is reviewed in depth elsewhere (Kanagy, 2009; Lurie, 2011; Baltzis et al., 2016).

Lucking et al. (2014) demonstrated increased cardiac output in CIH-exposed rats without significant changes in femoral vascular conductance (Lucking et al., 2014). This study demonstrated unaltered vascular conductance in response to lumbar sympathetic stimulation in CIH-exposed rats. Aortic compliance was increased and estimated blood volume was unchanged in CIH-exposed rats. Increased blood pressure was related to an increase in cardiac output, which was confirmed by echocardiography (Lucking et al., 2014). It is suggested therefore that hypertension in the CIH model can be evoked by over-excitation of the cardiac arm of sympathetic nervous system (SNS), even before mechanisms of enhanced peripheral vasoconstriction and endothelial dysfunction are initiated (Naghshin et al., 2009). This may have less relevance to human OSAS where increased cardiac output is not typically observed. Indeed, cardiac fibrosis, inflammation and apoptosis were reported in CIH-exposed animals, revealing long-term deleterious outcomes for the CIH-exposed heart (Wei et al., 2016).

### Carotid Body Response to CIH

Disturbances in the autonomic nervous system alter cardiac output and vascular tone, which can result in blood pressure elevation. This raises the issue of the cause of autonomic disturbance in CIH models and OSAS. It is possible that the carotid bodies and kidneys play a role in driving sympathoexcitation. Both organs are sensitive to modest decreases in the partial pressure of oxygen (Patinha et al., 2017) and are innervated by afferent nerves that project to hindbrain regions, which play a key role in modulating sympathetic outflow (Johns et al., 2011; Patinha et al., 2017). Carotid bodies, known as peripheral chemoreceptors, are clusters of cells located in the bifurcation of the carotid artery. They are composed of type I glomus cells and type II glial cells. Type I cells respond to an impairment in blood flow and mainly to decreasing levels of arterial PO_2_ (Andrade et al., 2018). The carotid body chemoreflex modulates sympathetic drive to the heart and vasculature to maintain blood pressure. Selective bilateral carotid body ablation resulted in systolic blood pressure reduction and a decrease in the respiratory response to hypoxia in CIH-induced hypertensive rats and spontaneously hypertensive rats (SHR) (Del Rio et al., 2016; Pijacka et al., 2018). However, carotid body ablation exaggerated the pressor response to hypercapnia in SHR rats, which might be due to blunted respiratory response and CO_2_ accumulation in the blood (Pijacka et al., 2018). In CIH-exposed animals, there is evidence of carotid body sensitization and exaggerated responsiveness (Prabhakar et al., 2010; Iturriaga et al., 2015; Shell et al., 2016). During hypoxia, a decrease in the partial pressure of oxygen in the blood causes a decrease in heme oxygenase 2 (HO-2) levels in the carotid bodies leading to a decrease in carbon monoxide production. Normally, carbon monoxide suppresses the production of dihydrogen sulfide (H_2_S). Therefore, hypoxia increases H_2_S levels which stimulates carotid sinus nerve firing (Shell et al., 2016). This leads to sympathetic over-excitation which causes oxidative stress in other peripheral organs such as the kidney (Kumar et al., 2015). Similar to renal sensory receptors, substance P (SP) release is implicated in carotid body excitation (Peng et al., 2011). Indeed, ablation of the carotid bodies prevents the development of CIH-induced hypertension (Prabhakar and Kumar, 2010). Carotid body ablation restored the normal bradycardia response to propranolol, which was significantly enhanced after 28 days of CIH exposure. In addition, heart rate variability was progressively exaggerated in CIH-exposed rats after the 7th day of exposure but was restored to normal following surgical ablation of the carotid bodies (Del Rio et al., 2016). In contrast, carotid body denervation partially attenuated the increase in sympathetic activity and heart rate observed after acute upper airway obstruction (Ferreira et al., 2018). Moreover, administration of 100% oxygen to silence the activity of carotid bodies during upper airway obstruction reduced, but did not eliminate the increase in sympathetic nerve activity. These results suggest that carotid body afferents partially contribute to sympathoexcitation during apnoea (hypoxic hypercapnia). Inhibition of NTS neurons reduced phrenic, renal, lumbar, and splanchnic nerve activity and heart rate during upper airway obstruction (Ferreira et al., 2018). However, the influence of central chemoreceptor inputs to SNS activity is likely important during upper airway obstruction as CO_2_ and hydrogen ions activate chemosensitive sites, which leads to sympathoexcitation and resultant cardiorespiratory changes.

Augmented carotid body responsiveness to acute challenges such as hypoxia or hypercapnia was demonstrated in CIH-exposed animals (Huang et al., 2009). In addition, stimulation of the carotid bodies using cyanide resulted in an exaggerated and prolonged sympathoexcitation represented by greater thoracic sympathetic nerve activity in CIH-exposed juvenile rats (Braga et al., 2006). The intensified sympathetic nervous response can be due to modulations at the level of the central nervous system (discussed below) or the peripheral nervous system i.e. potentiated chemoreflexes. A growing body of evidence suggests enhanced basal afferent signaling from glomus cells of the carotid bodies under resting conditions after exposure to CIH, known as sensory long-term facilitation (sLTF) (**Figure 2**) (Peng et al., 2009). Previous studies have shown that sLTF is induced by the enhanced release of 5-hydroxytreptamine (5-HT) in carotid bodies. 5-HT binds to 5-HT_2_ receptors, which activates NOX enzymes causing ROS generation (Peng et al., 2009; Prabhakar et al., 2010). ROS upregulate ET-A receptors in the carotid bodies and potentiate carotid body responses to hypoxia (Pawar et al., 2009). Moreover, it was found that sLTF is induced by a NOX-superoxide signaling pathway dependent on overexpression of angiotensin 1 (AT1) receptors and Ang II in the carotid bodies (Marcus et al., 2010). Losartan, apocynin and AT1a receptor knockout abolished Ang II-mediated sLTF (Peng et al., 2011). Subsequently, sensitization of the carotid bodies by Ang II causes a further increase in renal sympathetic nerve activity (RSNA), which exaggerates RAAS activity and renal afferent signaling. Therefore, there is a possibility that persistent activity of the carotid body is secondary to sympathoexcitation, RAAS system activation and/or altered renal afferent chemosignals. Although Ang II and 5-HT induce sLTF of the carotid bodies, there is evidence that both are involved in the initiation, but not the maintenance of sLTF. Roy et al. (2017) discovered that transient receptor potential vanilloid 1 (TRPV1) channels are important for the maintenance of sLTF observed in carotid sinus nerve recordings from isolated perfused carotid body preparations using an acute intermittent hypoxic hypercapnic model (Roy et al., 2017). In addition, there is evidence of a contribution by ATP and P2X receptors in hypoxia-dependent carotid body activation (Moraes et al., 2018). P2X3 antagonism reduced hypoxic ventilatory responses in healthy rats, and carotid sinus nerve responses to hypoxia were attenuated in P2X2-deficient mice (Moraes et al., 2018). Recently, a potential role for sites beyond and independent of the carotid body in CIH-induced hypertension was revealed in studies of IH exposure in guinea-pigs, which have hypoxia-insensitive carotid bodies (Docio et al., 2018; Lucking et al., 2018). Exposure of guinea-pigs to a severe protocol of CIH (30 cycles/hr/day for 30 days) was associated with blood pressure elevation, an increase in plasma NE levels and an increase in heart rate (Docio et al., 2018). In contrast, exposure of guinea-pigs to modest CIH (6 cycles/h/day, 12 days) did not cause hypertension, but was associated with altered autonomic control of the heart and altered respiratory timing (Lucking et al., 2018). Together the studies suggest a primary role for carotid body sensitization in the elaboration of CIH-induced hypertension, but also convincingly reveal a capacity for cardiorespiratory impairment and the development of hypertension in the absence of carotid body plasticity. Among several potential mechanisms of action, it is plausible that the kidneys are implicated in CIH-induced autonomic dysfunction and hypertension as discussed in sections below.

### Higher Brain Centers and NTS Plasticity

Inspiratory and expiratory neurons of the brainstem form inhibitory and excitatory synaptic connections within the rostral ventrolateral medulla (RVLM), the site of the pre-sympathetic nerves. These connections generate respiratory-related signatures on sympathetic nervous outflow shaping the arterial pressure waveform, known as Traube-Hering waves (Moraes et al., 2012). The impact of respiration on arterial blood pressure relates to stimulation of lung stretch receptors and cyclical changes in the intra-thoracic pressure, which alter stroke volume and arterial baroreflex control, or modulation of the central coupling between respiratory complexes and sympathetic neurons in the ventrolateral medulla (VLM). In healthy rats at rest, post-inspiratory neurons in the BÖtzinger complex (BÖtC) inhibit expiratory neurons located in retrotrapezoid nucleus/parafacial respiratory group (RTN/pFRG). This renders expiration a passive process with typically no sympathetic nervous activity expressed in the late expiration phase (E2) (Machado et al., 2017). In CIH-exposed rats, Zoccal et al. (2008) reported increased forced expiratory activity in juvenile male rats after 10 days of exposure to CIH along with a distinctive discharge of abdominal nerve activity during the E2 phase of ventilation. In this phase, thoracic sympathetic neuronal activity was greater than in sham rats (Zoccal et al., 2008). It was suggested that the expiratory neurons in the BÖtC of the ventral respiratory column (VRC) suppress the activity of post-inspiratory neurons on the basis of observed decreases in cranial vagus nerve activity in the post-inspiratory phase (Zoccal et al., 2008). This suppression augments the activity of expiratory neurons and alters excitability of the RVLM neurons. In addition, post inspiratory neurons normally activate GABAergic inhibitory neurons of the caudal venterolateral medulla (CVLM). Therefore, suppression of post inspiratory neurons depresses the inhibitory CVLM activity, which results in the excitation of RVLM presympathetic neurons (Zoccal et al., 2009b). Spectral power analysis of arterial blood pressure of juvenile CIH-exposed rats showed a significant increase in the high frequency and low frequency powers as well as greater variable oscillations of arterial blood pressure, such that CIH-exposed rats exhibit larger Traube-Hering waves (Zoccal et al., 2009a; Moraes et al., 2012). This indicates an excessive sympathoexcitation and enhanced respiratory modulation of sympathetic nervous activity during expiration in juvenile CIH-exposed animals (Zoccal et al., 2009a).

Barnett et al. (2017) suggested the presence of a direct connection between second order neurons of the NTS, which receive peripheral chemoreceptors inputs, and central chemoreceptors of the RTN. Excitatory inputs from peripheral chemoreceptors activate late expiratory neurons of the RTN in the CIH model. This in turn enhances sympathetic discharge from the brainstem in the late expiratory phase (Barnett et al., 2017). Molkov et al. (2011) suggested that exposure to CIH increases the sensitivity of the central chemoreceptors located in the RTN/pFRG to CO_2_ levels (Molkov et al., 2011). During hypercapnia, RTN/pFRG excites the expiratory neurons located in the caudal VRC, which causes an enhanced discharge of abdominal nerves during late expiration (Molkov et al., 2011). This is similar to the enhanced abdominal nerve activity seen in CIH animals during late expiration under normal levels of CO_2_ (Zoccal et al., 2008). This suggests an interaction between RTN/pFRG chemoreceptors with late expiratory neurons and presympathetic nerves of the RVLM, which elaborates an increase in thoracic sympathetic nerve activity (Molkov et al., 2011). It is also suggested that peripheral chemoreceptors increase the excitability of pre-inspiratory neurons of the pre-BÖtC complex. Excessive excitatory inputs from pre-inspiratory neurons to the late expiratory neurons in the RTN provides an additional E2 activity even under normal levels of CO_2_ (Barnett et al., 2017). On the other hand, Souza et al. (2016) reported an enhanced sympathetic outflow during inspiration in juvenile female rats exposed to CIH with no changes in the expiratory activity (Souza et al., 2016). It is proposed that enhanced sympathetic discharge mediated by altered respiratory modulation presenting cyclically causes more pronounced vasoconstriction and blood pressure elevation compared with sustained nervous activity, a mechanism that may contribute to CIH-induced hypertension (Souza et al., 2016).

Immunohistochemical studies revealed a 20% increase in the expression of P2X3 and P2X4 receptors in the RVLM region after 10 days of exposure to CIH. ATP injection in these animals resulted in larger increases in thoracic sympathetic nerve activity compared with sham animals (Zoccal et al., 2011). Additionally, a previous study reported an increase in the expression of N-methyl-D-aspartate (NMDA) and AMPA receptors in the caudal region of the NTS (cNTS), which is the main site for integration of chemoreceptors inputs. This explains the enhanced sympathetic activity after glutamate injection in the cNTS in CIH-exposed rats compared with healthy rats (Costa-Silva et al., 2012). Other studies showed that exposure to CIH is associated with the upregulation of glutamatergic neurons in the NTS and increased expression of c-fos and fosB in the RVLM (Oyarce and Iturriaga, 2018). Therefore, increased peripheral afferent activity causes exaggerated central neural activity, which increases the production of ROS and cytokines in the NTS. This induces microglial activation, which stimulates further increases in the levels of inflammatory cytokines. Exposure to CIH for 21 days caused an elevation of pro-inflammatory cytokines in the NTS and RVLM (Oyarce and Iturriaga, 2018). This hyperactivates NTS and PVN neurons, which increases renal sympathetic nerve activity and Ang II secretion. Conversely, continuous afferent neural activity causes a depression of NTS neurotransmission. Indeed, in CIH-exposed animals there is depression of glutamate-dependent neurotransmission and a decrease in the number of NTS active synapses (Almado et al., 2012). This alters the integration of sensory inputs including baroreceptors and chemoreceptors, which might contribute to sustained sympathoexcitation even during normoxia (Kline, 2010).

Neural activity of the hypothalamic paraventricular nucleus (PVN) is controlled by excitatory neurotransmitters such as glutamate and inhibitory neurotransmitters including NO. In normal healthy rats, the PVN represents the central regulator that controls renal sympathetic discharge and blood pressure regulation (McBryde et al., 2018). Endogenous nNOS knockout selectively in the PVN of healthy Wistar rats resulted in 70% increase in RSNA with a significant gradual increase in blood pressure reaching a plateau after 10 days (McBryde et al., 2018). Exposure to IH for 14 days resulted in a decrease in plasmalemmal density of NMDA NR1 in n-NOS containing dendrites with a reduction in NMDA-evoked currents in the PVN prior to the onset of blood pressure elevation (Coleman et al., 2010). Nevertheless, NO production was not altered after 14 days of exposure to IH (Coleman et al., 2010). Of interest, blood pressure elevation after exposure to a different protocol of CIH was not associated with changes in nNOS expression or NO production in the NTS region (Pajolla et al., 2009). In contrast, prolonged exposure to CIH (35 days) caused an accumulation of NMDA NR1 in the cytoplasm of n-NOS containing dendrites in addition to suppression of NO production after the onset of hypertension (Coleman et al., 2010). Importantly, GABA-containing and nNOS-containing interneurons of the PVN region receive afferent inputs from the NTS that mediate arterial baroreflex control of blood pressure (Affleck et al., 2012; Abdulla and Johns, 2013, 2014). Therefore, this supports the possibility that the normal regulatory baroreflex might be dysregulated or retuned after exposure to CIH, an important issue under investigation with variable answers in literature (discussed further below in “Renal oxidative stress, inflammation and CIH-induced hypertension”).

### Kidney Response to CIH

Exposure to CIH and resultant decreases in renal oxygenation stabilize the HIF-1α subunit (Haase, 2006). HIF-1 is a transcription factor composed of a hypoxia-sensitive alpha subunit and a constitutive beta subunit. HIF-1 drives the expression of genes that encode erythropoietin, heme oxygenase 1 (HO-1) and vascular endothelial growth factor (VEGF), responsible for fibrosis and angiogenesis (Haase, 2006; Sun et al., 2012; Lu et al., 2017b; Abuyassin et al., 2018) (Table 2). Erythropoietin increases red blood cell production and oxygen carrying capacity as indicated by increased haematocrit in CIH-exposed animals (Saxena et al., 2015). O'Neill et al. (2019) recently described persistent reductions in renal cortical oxygenation following exposure to long-term, but not short-term IH. The kidneys are also affected by sensitization of the carotid bodies to hypoxia. The exaggerated response of the carotid bodies results in an increase in RSNA, which stimulates the release of catecholamines from the adrenal medullae and activates the RAAS. As part of this response, there is increased vasoconstriction and enhanced renal tubular sodium and water reabsorption leading to increased renal metabolic demand. Table 2 summarizes a number of studies in CIH models whereby decreased blood oxygenation was associated with oxidative stress and inflammation in renal tissues. Inflammatory metabolites trigger excitatory sensory responses mediated by renal chemoreceptors, which signal to the NTS and RVLM brain regions. This results in excessive activation of sympathetic nervous outflow, which causes further RAAS activation in a vicious cycle, as in the case of renal ischemic reperfusion injury (Cao et al., 2017). RAAS activation induces Ang II release, which stimulates the circumventricular organs (CVO) of forebrain nuclei such as the SFO and median preoptic nucleus (MnPO) (Sharpe et al., 2013; Saxena et al., 2015). Activation of CVO regions in turn stimulates PVN and contributes to sustained sympathetic outflow (Sharpe et al., 2013). Knockout of AT1a receptors of the SFO attenuated blood pressure and FosB staining in PVN and MnPO of CIH-exposed animals (Saxena et al., 2015).

**Table 2:** Oxidative stress and inflammatory biomarkers (↑, an increase; ↓, a decrease) in animals exposed to different protocols of CIH.

**Biomarker**	**Level**	**Sample**	**Duration of exposure to IH (days, h/day)**	**Species**	**Frequency of exposure (cycles/h)**	**Intensity of hypoxia (% of O_**2**_)**	**References**
8-hydroxyl deoxy-guanosine	↑	Urine	28 days, 8 h/day	Mice	12	10%	Keiko et al., 2017; Snyder et al., 2017
Apoptotic cells	↑	Kidney tissue	56 days, 12 h/day	Mice	30	8%	Sun et al., 2012; Ding et al., 2016; Wu X. et al., 2016; Lu et al., 2017a,b; Zhang X. B. et al., 2018; Zhang Y. et al., 2018
	↑	Kidney tissue	21 days, 8 h/day	Rats	40	5%	
			28 days, 8 h/day				
	↑	Kidney tissue	112 days, 8 h/day	Rats	30	5–6%	
	↑	Kidney tissue	35 days, 8 h/day	Rats	60	6–7%	
	↑	Kidney tissue	42 days, 8 h/day	Mice	30	6–8%	
			84 days, 8 h/day			5–7%	
Bax/Bcl-2	↑	Kidney tissue	35 days, 8 h/day	Rats	60	6–7%	Wu X. et al., 2016; Abuyassin et al., 2018; Zhang Y. et al., 2018
	↑	Kidney tissue	60 days, 12 h/day	Mice	60	8%	
	↑	Kidney tissue	42 days, 8 h/day	Mice	30	6–8%	
Caspase 3	↑	Kidney tissue	42 days, 8 h/day	Mice	30	6–8%	Zhang X. B. et al., 2018; Zhang Y. et al., 2018
			84 days, 8 h/day			5–7%	
Catalase	↓	Kidney tissue	28 days, 8 h/day	Rats	40	5%	Lu et al., 2017b
Collagen 1	↑	Kidney tissue	56 days, 12 h/day	Mice	30	8%	Wu et al., 2015; Zhang Y. et al., 2018
			42 days, 8 h/day	Mice	30	6–8%	
Collagen IV	↑	Kidney tissue	42 days, 8 h/day	Mice	30	6–8%	Zhang Y. et al., 2018
Connective tissue growth factor (CTGF)	↑	Kidney tissue	56 days, 12 h/day	Mice	30	8%	Sun et al., 2012; Wu et al., 2015; Lu et al., 2017b; Abuyassin et al., 2018
	↑	Kidney tissue	28 days, 8 h/day	Rats	40	5%	
	↑	Kidney tissue	21 days, 12 h/day	MT knockout mice	30	8%	
	↑	Kidney tissue	60 days, 12 h/day	Mice	60	8%	
ERK1/2 phosphorylation	↑	Kidney tissue	3 days, 14 days and 56 days, 12 h/day	Mice	30	8%	Sun et al., 2012; Wu X. et al., 2016
	↑	Kidney tissue	35 days, 8 h/day	Rats	60	6–7%	
HIF-1α	↑	Kidney tissue	56 days, 12 h/day	Mice	30	8%	Sun et al., 2012; Lu et al., 2017b; Poonit et al., 2017; Abuyassin et al., 2018
	↑	Kidney tissue	28 days, 8 h/day	Rats	40	5%	
	↑	Kidney tissue	14 and 28 days, 7.5 h/day	Rats	40	9%	
	↑	Kidney tissue	56 days, 12 h/day	Mice	60	8%	
ICAM-1	↑	Kidney tissue	3 days, 7 days and 56 days, 12 h/day	Mice	30	8%	Sun et al., 2012; Wu et al., 2015
IFN-γ, IL-10, IL-13, IL-2, IL-4, IL-5	↓	RVLM	7 days, 8 h/day	Mice	10	10%	Snyder et al., 2017
IL-1β	↓	RVLM	7 days, 8 h/day	Mice	10	10%	Snyder et al., 2017
	↑	Kidney tissue	NA, 10 h/day	Mice	60	7%	Wu et al., 2018
IL-6	↑	Kidney tissue, Plasma	28 days, 8 h/day	Rats	40	5%	Wu X. et al., 2016; Lu et al., 2017b; Snyder et al., 2017; Zhang Y. et al., 2018
	↑	Serum	35 days, 8 h/day	Rats	60	6–7%	
	↓	RVLM	7 days, 8 h/day	Mice	10	10%	
	↑	Kidney tissue	42 days, 8 h/day	Mice	30	6–8%	
JNK phosphorylation and P38 phosphorylation	↑	Kidney tissue	35 days, 8 h/day	Rats	60	6–7%	Wu X. et al., 2016
Kidney morphology	No change (Hematoxylin and Eosin)	Kidney tissue	3 days, 7 days, 21 days and 56 days, 8 h/day	Mice	30	8%	Wu et al., 2015, 2018; Lu et al., 2017b; Poonit et al., 2017; Zhang X. B. et al., 2018; Zhang Y. et al., 2018
			84 days, 8 h/day			5–7%	
	No change (Hematoxylin and eosin)	Kidney tissue	28 days, 8 h/day	Rats	40	5%	
	Glomeruli dilatation and hypertrophy of epithelial cells of tubules	Kidney tissue	14 and 28 days, 7.5 h/day	Rats	40	9%	
	Tubular vacuolization, tubular epithelial cell exfoliation, inflammatory cell infiltration, and thickening of glomerular basement membrane	Kidney tissue	NA, 12 h/day	Mice	60	7%	
	Tubular atrophy	Kidney tissue	42 days, 8 h/day	Mice	30	6–8%	Zhang Y. et al., 2018
Liver-type fatty acid-binding protein (L-FABP)	↑	Urine	28 days, 8 h/day	Mice	12	10%	Keiko et al., 2017
Malondialdehyde	↓	Kidney tissue	3 days and 14 days, 12 h/day	Mice	30	8%	Xiang et al., 2010; Sun et al., 2012; Wu et al., 2015, 2018
	↑	Kidney tissue	56 days, 12 h/day	Mice	30	8%	
	↑	Kidney tissue	7 days, 12 h/day	Mice and methionine (MT) knockout mice	30	8%	
	↑	Serum	42 days, 7 h/day	Rats	20	6–8%	
	↑	Kidney tissue	NA, 10 h/day	Mice	60	7%	
MCP-1	↑	Kidney tissue	42 days, 8 h/day	Mice	30	6–8%	Zhang Y. et al., 2018
Metallothionen	↑	Kidney tissue	3 days and 7 days, 12 h/day	Mice	30	8%	Wu et al., 2015
	↓	Kidney tissue	14 and 56 days, 12 h/day	Mice	30	8%	Sun et al., 2012; Wu et al., 2015
Mn SOD and Cu/Zn SOD	↓	Kidney tissue	14 days and 28 days, 7.5 h/day	Rats	40	9%	Poonit et al., 2017
NADPH dehydrogenase	↑	Kidney tissue	3 days and 7 days, 12 h/day	Mice	30	8%	Wu et al., 2015
	↓		56 days, 12 h/day	Mice			
NF-κB	↑	Serum	35 days, 8 h/day	Rats	60	6–7%	Wu X. et al., 2016
Nitrotyrosine and caspases	↑	Kidney tissue	28 days, 8 h/day	Rats	40	5%	Lu et al., 2017b
Nrf2 and HO-1	↑	Kidney tissue	3 days, 12 h/day	Mice	30	8%	Sun et al., 2012; Wu et al., 2015
	Normal	Kidney tissue	56 days, 12 h/day	Mice			
	↑	Kidney tissue	3 days, 7 days and 21 days, 12 h/day	Mice			
				MT knockout mice			
	↓	Kidney tissue	56 days, 12 h/day	Mice			
				MT knockout mice			
Oxidative low- density lipoproteins	↑	Serum	42 days, 7 h/day	Rats	20	6–8%	Xiang et al., 2010
Oxidized cysteine (CysSSP)	↑	Kidney tissue	14 days, 10.5 h/day	Rats	5.6	5%	Coelho et al., 2018
Plasminogen activator inhibitor-1 (PAI-1)	↑	Kidney tissue	3 days, 7 days and 56 days, 12 h/day	Mice	30	8%	Sun et al., 2012; Wu et al., 2015
	↑	Kidney tissue	7 days, 12 h/day	MT knockout mice			
Reduced cysteine (CysSH)/CysSSP	↓	Kidney tissue (medulla)	14 days, 10.5 h/day	Rats	5.6	5%	Coelho et al., 2018
		Kidney tissue (cortex)	21 days, 10.5 h/day				
Renal fibrosis (Sirius red stain)	↑	Kidney tissue	14 days, 8 h/day	Rats	40	5%	Lu et al., 2017b
	↑	Kidney tissue	56 days, 12 h/day	Mice	30	8%	Sun et al., 2012; Wu et al., 2015
ROS	↑	Kidney tissue	112 days, 8 h/day	Rats	30	5–6%	Ding et al., 2016
SOD	↑	Kidney tissue	21 days, 8 h/day	Rats	40	5%	Lu et al., 2017a,b; Poonit et al., 2017; Wu et al., 2018
	↓	Kidney tissue	28 days, 8 h/day	Rats	40	5%	
	↓	Serum	14 and 28 days, 7.5 h/day	Rats	40	9%	
	↓	Kidney tissue	NA, 10 h/day	Mice	60	7%	
TGF-α	↑	Kidney tissue	28 days, 8 h/day	Rats	40	5%	Lu et al., 2017b
TGF-β	↑	Kidney tissue	28 days, 8 h/day	Rats	40	5%	Wu et al., 2015; Lu et al., 2017b; Abuyassin et al., 2018
	↑	Kidney tissue	56 days, 12 h/day	Mice	30	8%	
	↑	Kidney tissue	21 days, 12 h/day	MT knockout mice	30	8%	
	↑	Kidney tissue	60 days, 12 h/day	Mice	60	8%	
TNF-α	↑	Kidney tissue, plasma	28 days, 8 h/day	Rats	40	5%	Wu X. et al., 2016; Lu et al., 2017b; Snyder et al., 2017; Zhang Y. et al., 2018
	↑	Serum	35 days, 8 h/day	Rats	60	6–7%	
	↓	RVLM	7 days, 8 h/day	Mice	10	10%	
	↑	Kidney tissue	42 days, 8 h/day	Mice	30	6–8%	
Vascular cell adhesion protein 1 (VCAM-1)	↑	Kidney tissue	7 and 56 days, 12 h/day	Mice	30	8%	Sun et al., 2012; Wu et al., 2015
	↑	Kidney tissue	7 days, 12 h/day	MT knockout mice			
VEGF	↑	Kidney tissue	60 days, 12 h/day	Mice	60	8%	Abuyassin et al., 2018

In recent years, there has been increased interest in the potential role played by renal nerves in mediating a response from the injured kidney leading to hypertension in the CIH model. Witkowski et al. (2011) observed a decrease in blood pressure after catheter-based renal denervation in OSAS patients. A significant decrease in systolic and diastolic blood pressure has been observed up to 6 months following surgery in human studies (Witkowski et al., 2011; Kario et al., 2016a). Similar findings were reported after renal denervation in a mouse model of exposure to IH for 4 weeks (Kario et al., 2016a; Keiko et al., 2017), where a decrease in blood pressure and an associated reduction in circulating NE and angiotensinogen levels were reported. It has also been shown that catheter-based renal denervation is effective in decreasing hypoxia-induced nocturnal blood pressure elevation (Kario et al., 2016b). Kidney perfusion with hypoxic blood increases femoral perfusion pressure in rabbits, which was abolished by renal denervation (Ashton et al., 1994). Renal denervation and anti-oxidant treatment significantly decreased renal oxidative stress and blood pressure in CIH models (Xiang et al., 2010; Keiko et al., 2017; Lu et al., 2017b).

Renal denervation or adrenal demedullation eliminates chronic hypertension in CIH-exposed rats (Bao et al., 1997), related to a decrease in epinephrine secretion shown in the same study. Epinephrine stimulates NE release from postganglionic neurons, which induces vascular constriction and serves as a stimulus for RAAS activation (Bao et al., 1997). In support of this, plasma renin activity was elevated four-fold following IH exposure and was restored to baseline levels by renal denervation. In addition, losartan administration eliminated blood pressure elevation after IH exposure (Fletcher et al., 1999), suggesting that hypoxia, through carotid body activation, enhances RSNA, and Ang II release. Moreover, hypoxia induces the release of ischaemic metabolites and kidney injury, which indirectly enhance RSNA and RAAS. Finally, denervation of the adrenal medullae reduces NE release and eliminates the stimulus that activates renal sympathetic nerves, which are part of the reno-renal reflex (Bao et al., 1997). Overall, this reveals the presence of signals originating from hypoxic/injured kidneys that cause over-excitation of RSNA and blood pressure elevation, which are at least partly attributed to renal oxidative stress. These studies point to an important role played by renal nerves in mediating hypertension in the CIH model. The exact mechanisms, however, are not established.

## Renal Sensory Afferent Nerves

Renal sensory receptors are present as free nerve endings in the kidney that project to thoracolumbar region of dorsal root ganglion primarily from T12 to L3 (Weiss and Chowdhury, 1998). Afferent fibers synapse with interneurons within the ipsilateral dorsal horn in laminae I and laminae III to V. They project to brain sites including NTS, RVLM, SFO and PVN (Johns et al., 2011). There is considerable interest in the role of renal afferents in the control of sympathetic outflow and regulation of the cardiovascular system.

The location of the renal sensory nerve fibers has been identified using highly specific wheat germ agglutinin horseradish peroxidase nerve tracing (Marfurt and Echtenkamp, 1991). Immunohistochemistry has also been implemented to explore renal sensory nerves by tracking calcitonin gene-related peptide (CGRP) and SP as specific neurotransmitters of sensory neurons (Gontijo and Kopp, 1994; Kopp et al., 2007; Kopp, 2011b; Mulder et al., 2013). In the kidney, the sensory fibers travel parallel to the renal vein, renal artery and ureter entering the kidney at the hilus. The nerve endings of these sensory fibers are predominantly found in the ureter and the muscular layer of the renal pelvic wall with some located in the uroepithelial layer of the renal pelvis (Marfurt and Echtenkamp, 1991). This is evidenced by the presence of SP in the space between the muscular layer and the epithelial cells of renal pelvic wall (Feng et al., 2008) indicating the presence of sensory nerves in the renal pelvis. Similarly, CGRP was identified in the renal pelvic wall in previous studies in rat and sheep (Booth et al., 2015b; Foss et al., 2015) and was utilized to examine the validity of renal denervation in these studies. Marfurt and Echtenkamp (1991) identified renal afferent nerves in the renal cortex by labeling these fibers with wheat germ agglutinin-horseradish peroxidase. However, labeling was not detected in the renal medulla or papilla. Immunolabeling by the same group indicated the presence of afferent fibers in the interlobular and arcuate arteries. Some of these afferent sensory fibers were stimulated when the renal artery and renal vein were obstructed (Booth et al., 2015a; Xu et al., 2015). Similarly, an elevation in renal venous pressure resulted in an increase in urinary sodium excretion and urine flow rate. This response was not attenuated when lidocaine, a local anesthetic, was injected into the renal pelvis (Kopp et al., 1985) providing evidence for the presence of sensory nerves in the renal vein separate from those present in the renal pelvis. In general, sensory nerve endings in the kidney are of two types: mechanoreceptors and chemoreceptors (Stella and Zanchetti, 1991). In rats, around 76% of afferent nerves are unmyelinated slow conducting axons while some 19% are thin myelinated and only 5% are rapidly conducting myelinated fibers (Knuepfer and Schramm, 1987).

Mechanoreceptors are sensitive to stretch induced by volume expansion (VE), capsaicin or any stimulus that increases SP and CGRP formation. However, a group of renal sensory nerves were found to be responsive to ischemia due to renal artery occlusion or due to cyanide but not to pelvic pressure elevation (Recordati et al., 1978). This suggests that these sensory nerves are sensitive to the presence of ischemic mediators rather than mechanical stretch and were therefore referred to as chemoreceptors. Ischemia sensitive receptors are termed R1 chemoreceptors that are silent in their resting conditions but trigger a slow response characterized by long duration action potentials and their signals are transported mainly through non-myelinated nerve fibers (Recordati et al., 1978). In contrast, R2 chemoreceptors are active in the resting state and are slowly-adapting insofar as their firing continues 15–20 min after death. They are activated by renal ischemia and by backflow of hypertonic or hyperosmotic urine (Recordati et al., 1980; Goodwill et al., 2017). Studies pointed toward a role for chemoreceptors in initiating an excitatory reno-renal reflex, which contributes to sympathoexcitation and blood pressure elevation (Calaresu et al., 1976; Recordati et al., 1978; Goodwill et al., 2017). To distinguish the role of mechanoreceptors from that of chemoreceptors, an increase in renal pelvic pressure over a physiological range (2.5–10 mmHg) was utilized to activate the mechanoreceptors while a solution of 450 mM NaCl was used to stimulate the chemoreceptors (Kopp et al., 1998b; Wainford and Frame, 2017). However, there is still a scarcity in the literature of studies that differentiate between mechanoreceptors and chemoreceptors in terms of structure and function. Indeed, more studies are required to identify the different roles of these receptors in the reflex regulation of kidney and cardiovascular functions.

## Activation of Renal Sensory Neurons and Reno-Renal Reflexes

Signaling pathways involved in sensory mechanoreceptor activation have been extensively studied by Kopp (2015) employing pharmacological agents and direct recording of renal afferent nerve signals in rats. The renal afferent and efferent nerve fibers were found to exist in close proximity to each other according to histological studies (Kopp et al., 2007; Kopp, 2011b). This points to an interaction between afferent and efferent sympathetic nerves that play a key role in the control of body water and sodium balance, modulated by dietary sodium intake. This interaction is also modified by NE, ET-1 and Ang II (Kopp et al., 2002a, 2003, 2011; Kopp, 2011b). The renal pelvic wall contains AT-1 and ET receptors; ET-A receptors are located in the smooth muscle cell layer of the pelvic wall, with some immunoreactivity in blood vessels, while ET-B immunoreactive fibers are found in the uroepethelial layer of the pelvis running close to CGRP-labeled nerve fibers (Kopp et al., 2003, 2009).

**Figure 1:**
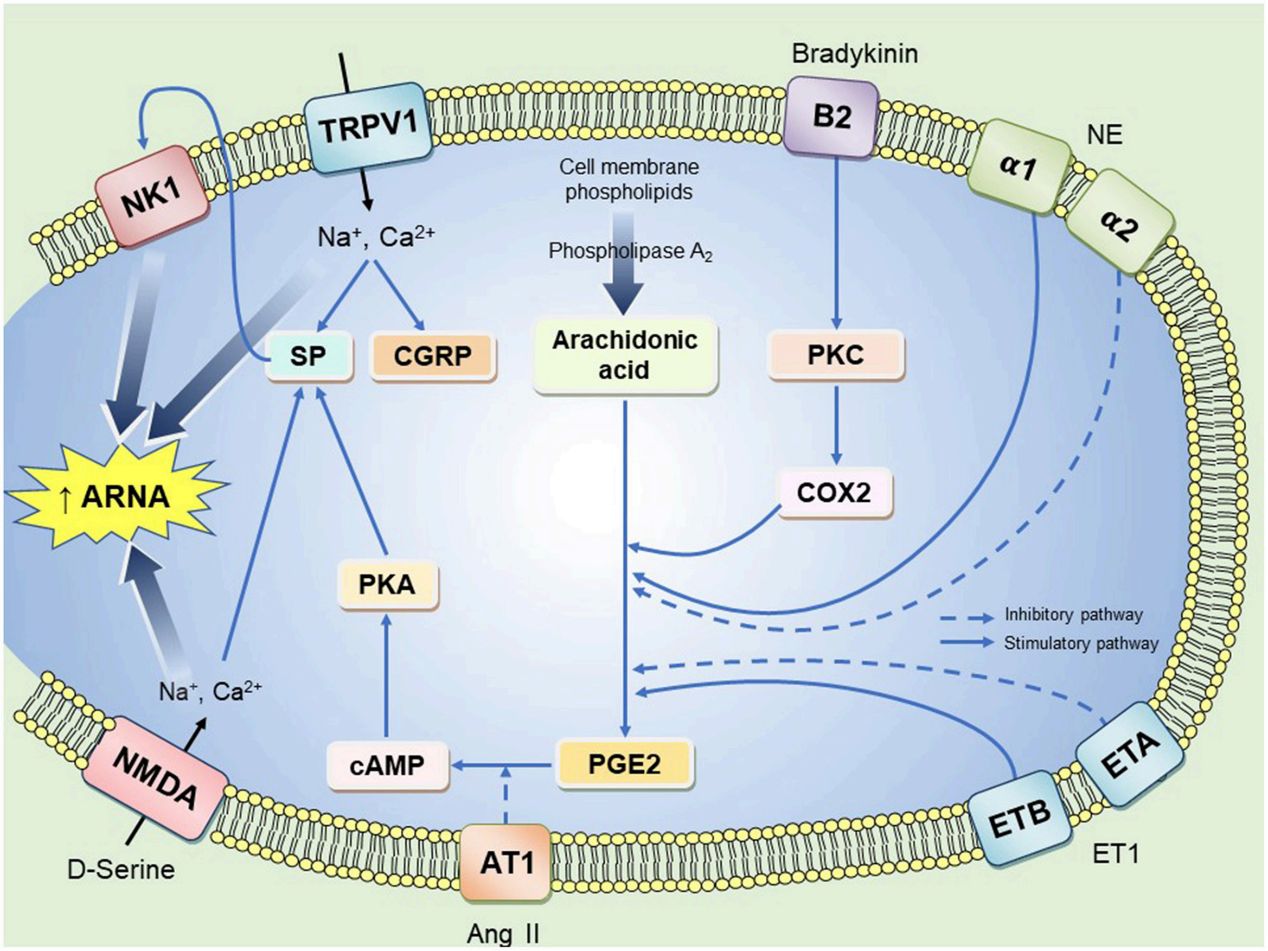
A representation of possible pathways that enhance renal afferent nerve activity in a renal afferent nerve ending. B2, Bradykinin receptor type 2; PKC, protein kinase C; NE, norepinephrine; ET1, endothelin 1; ETA and ETB, endothelin receptor type A and type B; PKA, phosphokinase A; SP, substance P; CGRP, calcitonin gene related peptide; NK1, neurokinin receptor 1; TRPV1, Transient receptor potential cation channel; NMDA, N-methyl-D-aspartate receptors; ARNA, afferent renal nerve activity. Solid lines represent excitatory pathways and dashed lines represent inhibitory pathways. Chronic intermittent hypoxia may evoke aberrant renal afferent signaling contributing to hypertension through action on one or more of the above signaling mechanisms.

Activation pathways of mechanoreceptors are via two main routes: neurokinin 1 receptor (NK1) activation by SP and/or stimulation of TRPV1 as illustrated in Figure 1. Some signaling pathways require the presence of cyclooxygenase (COX) enzyme and prostaglandins (PGs) as previously reported (Kopp and Smith, 1993). This explains why there is suppression of afferent renal nerve activity (ARNA) in rats treated with a COX-2 inhibitor or when their diet is deficient of essential fatty acids required for PG synthesis (Kopp and Smith, 1993; Kopp et al., 2000). The pathway that involves PGE2 binding to its receptors results in the activation of the cAMP/PKA signaling cascade that ultimately results in SP and CGRP production (Kopp et al., 2002b). This was further confirmed by immunohistochemical studies showing the co-localization of CGRP and PG receptors in sensory nerve terminals of the renal pelvic wall (Kopp et al., 2004). It should also be noted that the effect of SP on renal sensory afferents is associated with increased natriuresis and diuresis. This natriuretic and diuretic response relates to two mechanisms. First, SP activates an inhibitory reno-renal reflex to decrease sympathetic outflow, thus, increasing water and sodium excretion. Second, SP induces pelvic contractions that facilitate urine movement into the bladder (Kopp et al., 1998a). The role of CGRP in renal sensory nerve endings is not extensively studied but there is convincing evidence that CGRP stimulates renal afferent nerves and increases renal sodium excretion (Gontijo and Kopp, 1994, 1999; Xie et al., 2008).

Efferent nerves traveling into the kidneys are sympathetic in nature and are mainly located in the corticomedullary region and to a lesser extent, in the renal pelvic wall. The renal efferent nerves are intertwined with afferent nerves in the same nerve fiber bundle (Kopp et al., 2007; Kopp, 2011a). Afferent nerves are major players in the autonomic system response leading to increased activity of sympathetic efferents and hypertension (Sharpe et al., 2013). Efferent sympathetic stimulation increases NE release, which binds to α_1_ and α_2_ adrenoreceptors in the renal pelvic wall (Kopp et al., 2007). NE stimulates renin release followed by RAAS activation, which results in increased renal sodium and water reabsorption (Guyton, 2010). This causes an increase in ureter and renal pelvic pressure that activates mechanosensitive receptors through the opening of TRPV1 and release of SP, which in turn enhances ARNA. Renal afferent nerve stimulation provides a negative feedback mechanism that causes reflex natriuresis and diuresis i.e., a fall in efferent renal sympathetic nerve activity (ERSNA) (Kopp, 2015). This is known as the inhibitory reno-renal reflex, which protects against sympathetic over-activity and blood pressure elevation.

## Dietary Sodium, Renal Sensory Receptors, and Reno-Renal Reflex

The effect of dietary sodium on the interaction between renal afferent and efferent nerves in the control of sodium homeostasis has been studied under different sodium loading states. Indeed, challenges to sodium balance presents a reno-renal reflex response characterized by afferent renal nerve activation and sympathoexcitation. For example, low sodium diet consumption was found to be associated with higher levels of NE and Ang II (Anderson et al., 1989; Shao et al., 2013). NE binds to α_2_ adrenoreceptors while Ang II binds to AT1 receptors on renal afferents nerve endings, and both inhibit the PGE2 pathway leading to suppressed ARNA (Kopp et al., 2003, 2011). This effect of Ang II is partly-mediated through ET-1 binding to ET-A receptors (Kopp et al., 2009). In support of this notion, it was found that ET-A receptors are overexpressed when RAAS is activated in rats fed a low sodium diet or in congestive heart failure (Kopp et al., 2010). Kopp et al. (2002a) also showed that low sodium diet increases the threshold for mechanoreceptor activation from 2.5 to 5 to 7.5 mmHg. This decrease in mechanoreceptor sensitivity to increased pelvic pressure causes suppression of the inhibitory reno-renal reflex, hence sympathoexcitation ensues resulting in the retention of more sodium.

Alternatively, when dietary sodium intake is high, an inhibitory reno-renal reflex decreases ERSNA with resultant enhanced sodium excretion. This is associated with activation of ET-B receptors, which stimulates PGE2-mediated effects on ARNA (Kopp et al., 2009). These findings suggest that ERSNA is inappropriately elevated if there is a suppression of the inhibitory reno-renal reflex.

## Other Renal Sensory Receptors and Their Role in Reno-Renal Reflexes

TRPV1 is a ligand gated non-selective channel, which can be activated by heat, cations and vanilloid compound derivatives. TRPV1 is found in sensory neurons in the upper part of the ureter and neurons between uroepithelial and the smooth muscle cell layer of the renal pelvis (Zhu et al., 2007; Feng et al., 2008). TRPV1 allows the influx of Na^+^, Mg^2+^ and Ca^2+^ leading to SP and CGRP release in sensory neurons (Figure 1). This was supported by immunoblotting studies showing that SP, CGRP and TRPV1 are co-localized in the same sensory nerve, which was suggested to have mechanosensitive characteristics (Xie et al., 2008). TRPV1 is also present in renal afferent chemosensitive receptors as ipsilateral infusion of capsaizipine, a selective TRPV1 antagonist was shown to block contralateral diuresis triggered by hypertonic saline injection (Zhu et al., 2007). When TRPV1 is stimulated either by its selective agonist capsaicin or by the compound resiniferatoxin, it was found that this initiates an inhibitory reno-renal reflex. This effect was attenuated when a NK1 receptor antagonist was injected into the renal pelvis (Xie et al., 2008) suggesting cross-talk between TRPV1 and NK1 receptors in renal afferent neurons. Different signaling cascades were found to be activated through NK1 receptors including phospholipase A, which in rat kidney epithelial cells was shown to increase arachidonic acid levels and activate cyclic-AMP/PKA signaling mechanisms (Steinhoff et al., 2014). Although NK1 receptors play a key role in renal afferent nerve activation, their exact intracellular signaling pathway in the renal pelvis wall is not fully understood.

In addition to TRPV1 effects, bradykinin is a pro-inflammatory mediator that has been extensively studied and shown to activate afferent renal nerves (Smits and Brody, 1984; Maggi et al., 1992; Barry and Johns, 2015; Hindermann et al., 2017). It was suggested that bradykinin excites the afferent renal nerves via activation of renal chemoreceptors (Ashton et al., 1994). Indeed, Kopp et al. (2000) demonstrated the presence of bradykinin B2 receptors on sensory nerve fibers. In general, bradykinin binds to its receptors and activates protein kinase C, phospholipase A2 and the release of arachidonic acid (Kopp et al., 2000; Johns et al., 2011). Notably, local inflammation of peripheral sensory neurons stimulates B2 receptors to phosphorylate TRPV1 channels by G_q/11_ dependent mechanism indicating cross-talk between these receptors (Steinhoff et al., 2014). Similarly, a previous report indicated that B1 receptors are upregulated in inflammatory diseases (Petho and Reeh, 2012).

Bradykinin causes different renal hemodynamic changes depending on the route of administration. Intra-renal bradykinin elevates RSNA, decreases natriuresis and diuresis in the contralateral kidney with an immediate increase in heart rate, blood pressure and vascular resistance. This effect is abolished by renal denervation or by ganglionic blockade (Smits and Brody, 1984; Barry and Johns, 2015; Abdulla et al., 2016). However, intravenous injection of the same dose of bradykinin did not induce any significant changes in blood pressure or heart rate (Foss et al., 2015), rather it produced mesenteric vasodilation (Smits and Brody, 1984). The decrease in vascular resistance due to bradykinin was suggested to be mediated by an endothelial-dependent mechanism (Hoagland et al., 1999).

In support of this, Abdulla et al. (2016) investigated the role of renal bradykinin receptors in the modulation of the baroreflex in a cisplatin-induced renal failure model. Blockade of B2 bradykinin receptors, but not B1 receptors, markedly restored arterial baroreflex regulation of blood pressure in renal failure rats. In contrast, B1 blockade enhanced the RSNA sympathoinhibitory response to VE, but blunted this response in healthy animals (Abdulla et al., 2016). This suggests a possible role of B1 receptors in mediating a signaling pathway under basal conditions that maintains the normal baroreflex mechanism. Similar to the effect of B2 blockade, injection of a specific TRPV1 blocker into the kidney of cisplatin-induced renal failure rats restored the baroreflex control of RSNA indicating an important contribution of TRPV1 in mediating an aberrant response from the injured kidney leading to blunted baroreflex control (Abdulla et al., Unpublished Data). Together, these studies support the presence of an excitatory reno-renal reflex that is activated with contributions from a number of receptors such as TRPV1 and bradykinin. Similarly, inflammatory mediators such as TNF-α and IL-6 were found to be highly expressed in the kidney in models of renal injury and hypertension including exposures to CIH (Hall et al., 2014; Wu X. et al., 2016; Lu et al., 2017b) indicating a possible role of these mediators in initiating a sympathoexcitatory response leading to impaired blood pressure homeostasis.

Other candidates such as NMDA receptors are expressed in the renal afferent neurons (Figure 1). Ma et al. (2008) reported that activation of these receptors by the amino acid D-serine or by an increase in intra-pelvic pressure initiates an inhibitory reno-renal reflex response by increasing ARNA. Although this study provided evidence for an important role of NMDA receptors in mediating an afferent renal nerve-dependent response, the exact signaling pathway through which this response is mediated under normal or diseased states is not fully understood.

## Renal Afferent Nerves and Hypertension

Dysregulated autonomic control of blood pressure is recognized as an important component in the pathogenesis of different models of hypertension including two-kidney one clip Goldblatt model (Kumagai et al., 1990), SHR (Janssen et al., 1989), high fat diet-induced hypertension (Khan et al., 2015), and CIH-induced hypertension (Zoccal et al., 2009a; Shell et al., 2016). In these models, it was reported that high blood pressure is associated with sympathetic over-activity leading to peripheral vasoconstriction, renin release, and sodium and water retention. In the CIH model for example, a neurogenic mechanism is activated early in the disease process and is dependent on increased sympathetic tone (Sharpe et al., 2013). It is not known, however, if renal afferent signaling has a contributory role in mediating increased sympathetic nerve activity in CIH and OSAS. Of interest and relevance, renal denervation was found to ameliorate hypertension in sleep apnoea syndrome (Witkowski et al., 2011; Kario et al., 2016a; Keiko et al., 2017). Further investigation of reno-renal mechanisms as potential contributors to hypertension in the various models is required.

The baroreflex is impaired in several models of hypertension including some CIH models (Lai et al., 2006; Yamamoto et al., 2013). Short-term changes in blood pressure normally stimulate arterial (high pressure) and to some extent the cardiopulmonary (low pressure) stretch-sensitive baroreceptors. These receptors relay afferent signals to the brainstem to modulate sympathetic and parasympathetic nervous output. The central mechanism involved in the modulation of the autonomic response is dependent on functional renin-angiotensin and NO systems (Abdulla and Johns, 2013, 2014). Indeed, failure of the baroreflex mechanism is associated with long-term elevation of resting sympathetic nervous activity, which eventually leads to hypertension (Hart and Charkoudian, 2014). Kidney injury, through a reno-renal reflex mechanism, causes a blunted baroreflex through excessive sympathetic over-activity, which eventually leads to blood pressure elevation. In different models of hypertension, it was reported that impairment of the normal baroreflex mechanism is secondary to afferent renal nerve activation. Renal afferent nerve hyperactivity is associated with derangement of reno-renal reflex control, contributing to blood pressure elevation by suppression of the inhibitory reflex and/or activation of the excitatory reflex. This notion is supported by studies showing that suppression of the reno-renal reflex is involved in the pathogenesis of hypertension in SHR while activation of the excitatory reflex is proposed to play a major role in renovascular hypertension and hypertension associated with chronic kidney disease and obesity (Wyss et al., 1986; Janssen et al., 1989; Campese and Kogosov, 1995; Khan et al., 2015).

In the SHR model, elevation of peripheral sympathetic nerve activity is eliminated when the injured kidney is denervated (Kopp et al., 1998a) with almost a 50% decrease in blood pressure observed 10 min after denervation. Denervation is associated with a decrease in renal cortical tissue NE (Gao et al., 2016). Therefore, it has been suggested that part of the blood pressure lowering effect of renal denervation is due to a decrease in NE release from the postganglionic efferent nerves. However, it is also suggested that the decrease in blood pressure following denervation is due to interruption of renal afferent neuronal signaling. Janssen et al. (1989) used selective renal afferent nerve denervation to demonstrate the significant influence of renal afferents on SNS activity and baroreflex sensitivity in SHR. Renal afferent denervation in the latter study did not elicit a blood pressure lowering effect, and had no effect on urine volume or sodium excretion. Relevant to these findings, an increase in renal pelvic pressure or an intra-pelvic injection of bradykinin neither caused an increase in ARNA, nor an increase in SP release (Kopp and Smith, 1996; Kopp et al., 1998a). Moreover, there was a decreased responsiveness of NK 1 receptors to SP in SHR (Kopp et al., 1998a). These studies provide evidence for suppression of the inhibitory reno-renal reflex in SHR, which contributes to sympathetic over-stimulation in this model. Therefore, it appears that the blood pressure lowering effect of renal denervation in the SHR model is due to interruption of ERSNA and not related to afferent nerves.

In another model of hypertension, the two-kidney one clip model of hypertension, the clipped kidney undergoes ischaemic damage compared with the non-clipped kidney. Renal denervation of the non-clipped kidney induced a decrease in sodium excretion. In contrast, renal denervation of the clipped kidney resulted in an increase in ipsilateral and contralateral natriuresis along with a fall in contralateral ERSNA (Kopp and Buckley-Bleiler, 1989). Likewise, dorsal rhizotomy followed by clipping of the renal artery resulted in a significant partial decrease in blood pressure in the one-kidney one clip model. However, contralateral dorsal rhizotomy of the nephrectomised kidney did not cause any change in blood pressure (Wyss et al., 1986). This confirms the presence of excitatory afferent signals from the clipped kidney that underwent ischaemic insult. Previous studies suggested that ischemia induces adenosine release, which in turn stimulates afferent nerves (Liem et al., 2002). It is possible based on these studies that adenosine, being an ischaemic mediator, is associated with the activation of renal chemoreceptors (Ashton et al., 1994). In the one-kidney one clip model, it was found that blood pressure attenuation following denervation was not accompanied by an increase in diuresis and natriuresis (Katholi et al., 1981). Thus, sodium retention is a feature in the development of hypertension in this model, but it does not contribute significantly to the mechanism of the decrease in blood pressure after denervation. Based on these studies, it is possible that blood pressure reduction is attributed to an interruption of renal afferents, in addition to the effect of removal of efferent nerves to the kidneys.

Studies of cisplatin-induced renal injury and a high-fat diet model of obesity in rats demonstrated that it is the excitatory reno-renal reflex that is dominant in these models, responsible for blunted baroreflex control of blood pressure (Khan et al., 2014, 2017). In obese rats, for example, baroreflex sensitivity of RSNA and heart rate was blunted, in addition to a significant decrease in reflex sympathoinihibiton normally elicited by activation of the cardiopulmonary receptors in response to VE (Armitage et al., 2012; Khan et al., 2015). The dysregulation of the baroreflex in cisplatin or high-fat diet models is attributed to renal inflammation that initiates excitatory afferent neural signals, a suggestion supported by the increase in renal and systemic inflammatory cytokines such as TNF-α and interleukin- 6 (IL-6) (Khan et al., 2017). Similarly, transforming growth factor β1 (TGF-β1) was found to be elevated in renal tissue of the cisplatin-induced injury model, which was abolished following bilateral renal denervation (Goulding and Johns, 2015). The interaction between the immune system response in renal injury and renal afferent signaling mechanisms was highlighted in a study of obese rats treated with tacrolimus, an anti-inflammatory agent (Khan et al., 2017). In this study, suppression of the immune system restored the low- and high-pressure baroreflex. In addition, the renal tissue levels of TNF-α and IL-6 were reduced in these rats. However, dorsal rhizotomy of renal nerves did not decrease blood pressure in obese dogs (Hall et al., 2000). A study by Ditting et al. (2016) suggested that renal afferent excitability is decreased in the presence of chemokines, in particular, by CXCL2 (Ditting et al., 2016). Therefore, the contribution of inflammation to the sensitivity of renal afferent nerves is still not fully understood. Together, the involvement of renal nerves in mediating the derangement of the baroreflex control of blood pressure in these studies was evident when bilateral renal denervation restored the sensitivity of low- and high-pressure baroreflexes to changes in blood volume and blood pressure, respectively.

The effect of renal deafferentation was also studied in the 5/6 nephrectomy model and was shown to attenuate hypertension and decrease NE turnover in the brain (Campese and Kogosov, 1995). Moreover, renal afferent denervation decreased forebrain Ang II, AT1 receptors, c-fos in RVLM and tyrosine hydroxylase levels. Similarly, at the level of the kidney, macrophage count, fibrosis markers and NOX expression were reduced following denervation (Cao et al., 2015). In deoxycorticosterone acetate (DOCA) hypertensive rats, renal denervation attenuated arterial blood pressure by 50% together with a decrease in plasma NE. Interestingly, the same effect was observed after selective afferent denervation using capsaicin. Importantly, renal afferent denervation was performed prior to DOCA initiation which supports a key role for afferent nerves in the *development* of hypertension in this model (Foss et al., 2015). In the latter study, renal afferent denervation caused a significant decrease in the renal content of CGRP, with no effect on renal NE levels. This suggests that the renal afferents play a greater role than renal efferent nerves in this anti-hypertensive effect. TRPV1 is present on renal afferent nerve endings and is reported to mediate an increase in afferent nerve activity via SP. DOCA salt treatment in TRPV1-null mice showed exaggerated renal damage compared with wild-type mice in terms albuminuria, glomerulosclerosis, tubulointerstitial fibrosis and macrophage infiltration, yet blood pressure was equivalent between the two groups (Wang et al., 2008). This suggests a potentially key role for TRPV1 in initiating a renal afferent nerve-dependent response leading to increased blood pressure.

Ischemic reperfusion injury is another disease model associated with elevated blood pressure and heart rate. In this model, oxidative stress and inflammatory cytokines are elevated in the systemic circulation, SFO, hippocampus, corpus callosum and cerebral cortex. Overexpression of NOX2 and NOX4 in the kidney, SFO and hippocampus was reported in this model (Simone et al., 2014; Karim et al., 2015; Cao et al., 2017). In a renal ischemia reperfusion injury model, intracerebroventricular administration of tempol, losartan, and intra-renal capsaicin decreased blood pressure, and renal and central oxidative and inflammatory mediators (Cao et al., 2017). Ma et al. (2002) reported an impaired release of SP and downregulation of NK1 receptors in the post-ischaemic kidney. In the latter study, selective stimulation of mechanoreceptors by an increase in intra-pelvic pressure blunted the inhibitory reno-renal reflex and impaired renal excretory function. It can be suggested from the above studies that ischemia as characterized by increased adenosine and NOX, activates chemoreceptors in the kidney, which contribute to the enhanced sympathetic activity seen in hypertension.

## Renal Oxidative Stress, Inflammation and CIH-Induced Hypertension

Inflammation plays a major role in the pathogenesis of CIH-induced hypertension as reported by a number of studies (Sun et al., 2012; Iturriaga et al., 2015; Lu et al., 2017b; Abuyassin et al., 2018). In response to oxidative stress, HIF-1 stimulates the translocation of nuclear factor kappa B (NF-κB), which increases the expression of genes that encode TNF-α, interleukin 1β (IL-1β) and IL-6 (Del Rio et al., 2012; Iturriaga et al., 2015; Oyarce and Iturriaga, 2018). Chronic treatment with ibuprofen decreased cytokine levels in CIH-exposed carotid bodies, and attenuated the enhanced ventilatory response to hypoxia and prevented the development of hypertension (Del Rio et al., 2012). An anti-inflammatory treatment was associated with a decrease in c-fos levels in the NTS indicating a role for cytokines in neurogenic hypertension, in agreement with a number of previous reports (Sriramula et al., 2008; Kang et al., 2009; Shi et al., 2010; Del Rio et al., 2012). However, ibuprofen failed to decrease 3-nitrotyrosine levels and did not impede the augmented sensitivity of the carotid bodies to hypoxia (Del Rio et al., 2012). Meanwhile, anti-oxidant treatment abolished enhanced responsiveness of peripheral chemoreceptors and the ventilatory response to hypoxia along with a decrease in cytokine levels in carotid bodies (Del Rio et al., 2012). Thus, it is likely that inflammation and increased cytokine levels are secondary to an oxidative stress mechanism in the vasculature associated with exposure to CIH. In support of this argument, the NLRP3 inflammasome was found to be activated in renal tissue of CIH-exposed animals, which is well-known to be stimulated by ROS (Wu et al., 2018). NLPR3 is involved in the activation of caspase-1 and maturation of IL-1β, which is found to be elevated in the kidney of CIH-exposed animals. It was suggested that miR-155 gene inhibition suppresses ROS generation responsible for NLRP3 activation and subsequently IL-1β generation (Wu et al., 2018). Similar increases in inflammatory and oxidative stress markers were reported in the kidneys of CIH-exposed animals as shown by a number of studies in the literature (Table 2).

The increase in ROS is associated with an increase in renal tissue hypoxia factors including HIF (Ding et al., 2016). It has been shown that renal denervation and anti-oxidant treatment significantly decrease renal oxidative stress and blood pressure in CIH models (Xiang et al., 2010; Keiko et al., 2017; Lu et al., 2017b). Exposure to IH for 28 days and for 8 weeks was accompanied by HIF-1 upregulation in renal tissue (Keiko et al., 2017; Lu et al., 2017b). HIF-1α overexpression was also observed in NTS and RVLM after exposure to CIH (Peng et al., 2014). HIF-1α mediates the transcription of erythropoietin to increase the oxygen carrying capacity of the blood and protects against tissue hypoxia (Haase, 2006). HIF-1 upregulation is a ROS-dependent process mediated by NOX. Lu et al. (2017a) reported NOX4 overexpression in the kidney after exposure to CIH accompanied by a decrease in SOD levels. RSNA and blood pressure were both reduced when a NOX4 inhibitor was administered (Lu et al., 2017a). In addition, treating CIH-exposed rats with N-acetyl cysteine restored renal tissue SOD levels and decreased RSNA and blood pressure (Lu et al., 2017a). Immunohistochemistry demonstrated the co-localization of TRPV1 channels, NOX4 and H_2_O_2_ in renal sensory neurons (Lin et al., 2015). TRPV1 was found to be sensitized by H_2_O_2_ administration leading to SP release in renal mechanoreceptors (Lin et al., 2015). The effect of H_2_O_2_ on TRPV1 was inhibited by catalase. Likewise, ROS were suggested to be involved in mediating TRPV1 signaling in peripheral sensory nerves as well as in carotid bodies (Kline, 2010; Linley et al., 2012). In addition, increased ROS was reported in the carotid bodies due to an HIF-1-mediated NOX2 upregulation (Kumar and Prabhakar, 2012). This increase in ROS was associated with enhanced sympathetic nervous response to hypoxia. We can therefore propose that ROS might sensitize TRPV1 and upregulate AT1 receptors leading to a modification in the afferent nerve activity similar to its role in evoking sLTF of the carotid bodies following exposure to CIH (Marcus et al., 2010). This is further supported by studies whereby an elevation of malonaldehyde and oxidative low-density lipoproteins in renal tissue was positively correlated with Ang II levels and systolic blood pressure elevation after exposure to CIH (Xiang et al., 2010, 2012).

Additionally, metallothionein (MT) knockout mice exhibited renal fibrosis after 3 weeks of exposure to IH, but 8 weeks of exposure was needed to induce fibrosis in wild-type mice (Wu et al., 2015). Furthermore, SOD was downregulated while NOX2 was overexpressed in the NTS and RVLM after 10 days of exposure to IH (Peng et al., 2014). When viewed together, these findings highlight an important interaction between ROS and sympathoexcitation either at the level of the peripheral organs (carotid bodies and kidneys) and/or within the higher control centers.

The role of oxidative stress in mediating the elevation in blood pressure in CIH can be further demonstrated by a study showing that elevated ROS centrally was associated with a decrease in NO levels in the brain (Xu et al., 2004). Normally, NO controls the responsiveness of arterial and cardiopulmonary receptors to blood pressure changes (Abdulla and Johns, 2013, 2014). Indeed, impaired baroreflex control was reported in CIH-exposed rats after 17 days of exposure to IH (Lai et al., 2006). Exposure to IH for 7 days was associated with a resetting of the arterial baroreflex but with no effects on baroreflex gain (Yamamoto et al., 2013). This agrees with previous reports that illustrated increased expression of pro-inflammatory cytokines in some brain regions, but not in the RVLM and NTS (Snyder et al., 2017). However, exposure to CIH for longer periods was associated with inflammation in the NTS and RVLM, which might underpin blunted baroreflex function observed during the later stages of exposure to CIH (Lai et al., 2006; Oyarce and Iturriaga, 2018). Bilateral carotid body ablation improved baroreceptor sensitivity in CIH-exposed rats; however, blunted baroreflex gain of heart rate was not restored (Del Rio et al., 2016). This suggests the presence of other aberrant afferent inputs to the NTS in CIH-exposed rats contributing to blunted baroreflexes. In contrast, Moraes et al. (2016) reported increased gain of the high-pressure baroreflex during expiration using the *in situ* rat preparation after 10 days of exposure to IH. This was associated with CIH-mediated respiratory modulation and enhanced inhibitory responses of RVLM presympathetic neuronal activity (Moraes et al., 2016). In patients with OSAS and hypertension, an impaired baroreflex response to hypotension induced by sodium nitroprusside was reported. Interestingly, a blunted baroreflex response was seen even when blood pressure is within the normal range (Carlson et al., 1996), suggesting that derangements present before the evolution of hypertension. A full understanding of high-pressure baroreflex function in CIH-exposed animals has not yet been achieved.

OSAS is a known inflammatory disease that activates inflammatory signaling molecules in the systemic circulation and kidney tissue, which can cause renal fibrosis and apoptosis as indicated by studies of CIH-exposed animals (Adeseun and Rosas, 2010; Wu X. et al., 2016; Lu et al., 2017a). Exposure to CIH is associated with elevated expression of the NF-κB transcription factor in renal tissue and inflammatory cytokines including TNF-α, IL-6 and IL-1β in renal and blood samples (Wu X. et al., 2016; Lu et al., 2017b; Snyder et al., 2017; Zhang Y. et al., 2018). Short-term exposure to IH was sufficient to increase intercellular adhesion molecule (ICAM) levels (Sun et al., 2012). In addition, mitogen activated protein kinases enhanced the phosphorylation of JNK, P38 and ERK1/2 in renal tissue after exposure to CIH (Wu X. et al., 2016). Chronic kidney disease is often diagnosed in patients with OSAS. Therefore, molecular mechanisms underlying OSAS-induced kidney damage are currently of interest. Renal histopathological damage and inflammatory cytokines levels were attenuated in NLRP3 inflammasome knockout mice (Wu et al., 2018). Moreover, NLRP3 knockout and toll-like receptor 4 (TLR-4) knockout mice had significantly decreased serum creatinine and blood urea compared with wild-type CIH-exposed mice (Zhang Y. et al., 2018). TLR-4 deficient mice expressed fewer renal macrophages, collagen, fibroblast accumulation, and pro-apoptotic protein Bax expression after exposure to CIH compared with CIH-exposed wild-type mice. Importantly, MyD88 and NF-κB p65 expression was significantly attenuated in CIH-exposed TLR-4 knockout mice. Subsequently, elevated cytokines such as IL-6, monocyte chemoattractant protein 1 (MCP-1) and TNF-α, observed in CIH-exposed animals were alleviated in TLR-4 deficient mice (Zhang Y. et al., 2018). In another model of renal injury/inflammation, the obese rat model, it has been shown that sympathoexcitation and blunted baroreflex regulation was associated with elevated renal tissue levels of cytokines such as TNF-α and IL-6 (Khan et al., 2017). Ten days of exposure to IH (6% O_2_ for 40 s every 9 min) in juvenile male rats was enough to cause hypertension and enhance the sensitivity of the sympathetic and parasympathetic baroreflex regulation of blood pressure (Zoccal et al., 2009a). Interestingly, this suppression of the baroreflex was abolished when renal denervation was performed or when the immune system was suppressed pharmacologically in obese rats (Khan et al., 2017). Therefore, the renal afferent nerves in this model appear to play a key role in mediating a response from the injured kidney leading to increased sympathetic nervous activity and blood pressure. It is suggested that TNF-α and other inflammatory mediators are involved in blunting of the baroreflex and blood pressure elevation in OSAS patients. Most importantly, renal denervation studies in humans with OSAS report a decrease in sympathetic over-activity and blood pressure indicating a potential role played by renal nerves in the hypertension of OSAS (Witkowski et al., 2011; Linz et al., 2012; Zhao et al., 2013). Although the importance of renal nerves in OSAS-related hypertension in humans is recognized and a decrease in blood pressure is observed in patients following renal denervation, the detailed mechanisms underpinning this effect remain to be carefully determined. Together, the findings from previous studies suggest a mechanistic link between oxidative stress, increased production of cytokines and neural signals originating from the kidney as a potential driver of CIH-induced hypertension.

Based on previous studies, it appears that exposure to CIH induces a temporal response such that short-term or mild IH initiates oxidative and inflammatory signaling during which cellular defense mechanisms are activated to elicit a compensatory response (Table 2). However, long-term or severe IH induces inflammation, apoptosis and a decrease in the levels of antioxidant and anti-inflammatory enzymes (de-compensatory response) (Wu et al., 2015; Ding et al., 2016; Wu X. et al., 2016; Keiko et al., 2017; Lu et al., 2017b; Poonit et al., 2017). Although some studies reported compensatory responses for up to 3 weeks of exposure to IH, other studies reported renal morphological damage and attenuation of antioxidant enzymes after just 2 weeks of exposure to IH (Poonit et al., 2017).

As shown in Table 2, short-term IH exposure (3 days) is sufficient to induce lipid peroxidation, release of adhesion molecules (ICAM-1) and fibrogenetic factors (Sun et al., 2012). This indicates that IH might induce early renal injury as indicated by morphological changes and fibrosis (Sun et al., 2012; Poonit et al., 2017). Contemporaneously, protective antioxidative enzymes are induced within 3 days of exposure to IH (Sun et al., 2012). This addresses another issue regarding the cause of hypertension in OSAS patients. First, exposure to CIH might induce oxidative stress and inflammation within the kidney, which causes HIF-1 release and sodium and water retention, modulating reno-renal reflexes. As a result, the kidney cooperates with the carotid bodies in the activation of SNS outflow, evoking hypertension. Second, exposure to CIH stimulates sympathetic activity via actions on the carotid bodies, which causes blood pressure elevation, NE and Ang II release. This leads to kidney damage, modulation of reno-renal reflexes with deleterious consequences for the long-term control of blood pressure. Herein, we introduce a hypothesis, with supporting evidence from a range of studies, suggesting a role for renal afferent signaling in CIH-induced hypertension, with relevance to human OSAS.

## Conclusions

In CIH, the renal nerves play a key role in mediating pathophysiological responses, which contribute to derangement of blood pressure control leading to hypertension. Renal denervation in hypertensive humans with OSAS leads to a reduction in sympathetic nervous activity and blood pressure. Whereas, renal efferent nerves are implicated in kidney injury and hypertension, renal afferent nerves also play an essential causal role in sympathetic over-excitation and blood pressure elevation in various models of renal injury and hypertension. Exploration of mechanisms underpinning aberrant renal afferent signaling in experimental models is an area worthy of further investigation, especially in CIH-induced hypertension models. It is still unknown whether aberrant renal afferent signals represent activation of the excitatory reno-renal reflexes as revealed in a renal failure model, or suppression of inhibitory reno-renal reflexes as reported in SHR. Downregulation of NK1 receptors has been observed after exposure to chronic hypoxia, but not IH (Iacobas et al., 2006). This points to suppression of the mechanosensitive-mediated inhibitory reno-renal reflex in the CIH model. However, this does not exclude the possibility of coincident excitatory reno-renal reflex elevation of blood pressure, mediated by renal chemoreceptors. Possible mediators of aberrant renal afferent responses include ET with differential effects on ET-A (inhibits ARNA) and ET-B receptors (activates ARNA) under physiological conditions (Kopp et al., 2009). Of note, ET-A receptors are upregulated in the renal medulla of CIH-exposed animals (Guo et al., 2013).

**Figure 2:**
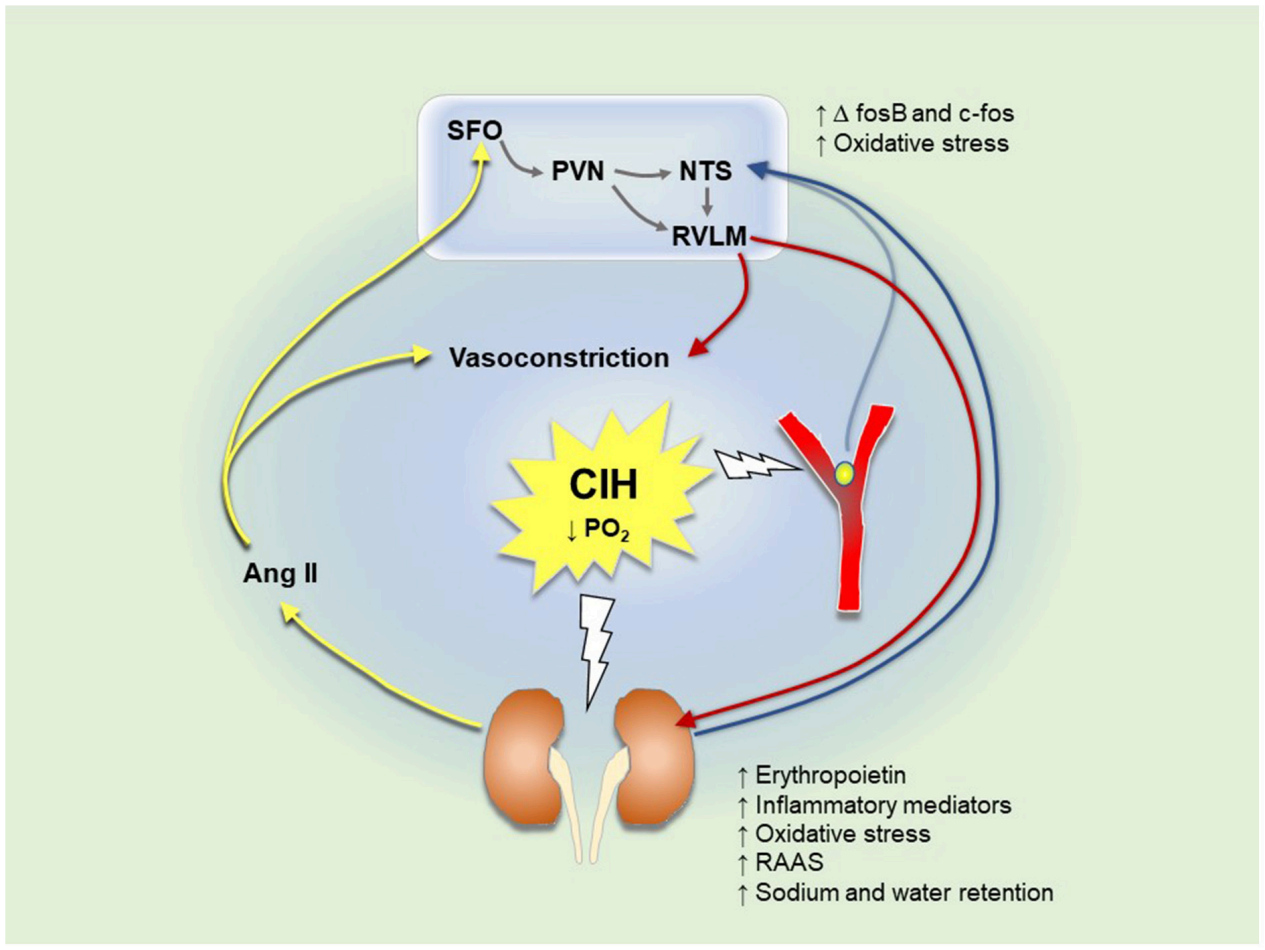
Mechanisms associated with the development of hypertension in chronic intermittent hypoxia (CIH)-exposed animals. Blue lines represent afferent signals; red lines represent efferent sympathetic discharge; yellow lines represent mechanisms involved in sensory long-term facilitation (sLTF). A decrease in the partial pressure of oxygen excites carotid body afferent discharge to the nucleus tractus solitarius (NTS). Signals are integrated to activate the rostral ventrolateral medulla (RVLM), increasing sympathetic nerve activity to different organs including the kidneys. An increase in efferent renal sympathetic nerve activity (ERSNA) activates the renin-angiotensin-aldosterone system (RAAS) and increases oxygen demand of the kidney. CIH also exerts direct and lasting effects on the kidneys. This stimulates afferent renal nerve signals, which are integrated in the NTS ultimately activating RVLM neurons, driving a reflex increase in ERSNA and additional release of angiotensin II (Ang II). Ang II causes vasoconstriction, which further decreases oxygen supply to the carotid bodies exaggerating their activity. Ang II stimulates circumventricular organs (CVO) such as the subfornical organ (SFO), which activates the paraventricular nucleus (PVN) of the hypothalamus enhancing sympathetic nerve activity. A decrease in renal oxygen levels is associated with increased secretion of erythropoietin. CIH elaborates a vicious cycle causing oxidative stress and inflammation in the carotid bodies, kidneys, and brain.

We acknowledge and emphasize that renal nerve-mediated responses are not the sole mechanism of hypertension in CIH, given the extensive literature revealing the importance of CIH-induced plasticity in the carotid bodies and central integrative sites, and respiratory-sympathetic coupling, which contribute to sympathetic nervous over-activity. There is convincing evidence that the development of hypertension in the CIH model is associated with sensitization of the carotid bodies. Hypertension is maintained even under normoxic conditions by a sLTF mechanism in the carotid bodies partly mediated by angiotensin II acting on AT1 receptors. Nevertheless, as evident by recent studies in guinea-pigs (Docio et al., 2018; Lucking et al., 2018), which have hypoxia-insensitive carotid bodies, other mechanisms are also suggested to play a role in the initiation and maintenance of CIH-induced hypertension, which we posit may be linked to decreased renal oxygenation and NTS plasticity (Figure 2).

Experimental protocols of CIH vary considerably (Table 2). Short-term exposure to IH causes the activation of cellular defense mechanisms and renal injury. This indicates an early role for the kidney in blood pressure regulation in disease models. Long-term exposure to IH induces the release of renal ROS and inflammatory cytokines, which modulate renal afferent signals to the NTS. Elevated renal ischaemic mediators can lead to renal chemoreceptor stimulation, with consequential activation of the excitatory reno-renal reflex. Exposure to CIH upregulates receptors such as ET-A receptors, which suppress the inhibitory reno-renal reflex. Similarly, TRPV1 and bradykinin receptors are recognized as important regulators of renal sensory signaling in different models of renal injury. As CIH is a known driver of renal injury and inflammation, the role of TRPV1 and other receptors on renal sensory nerve endings represents an important area for future research to widen our understanding of the pathogenesis of hypertension in models of CIH-induced hypertension, with implications for human sleep-disordered breathing.

## Author Contributions

SA wrote the manuscript. MA and KO edited and revised and manuscript. All authors revised and approved the final version.

### Conflict of Interest Statement

The authors declare that the research was conducted in the absence of any commercial or financial relationships that could be construed as a potential conflict of interest.

## Abbreviations

5-HT: 5-hydroxytryptamine
AHI: Apnoea-hypopnoea index
Ang II: Angiotensin II
ARNA: Afferent renal nerve activity
AT-1: Angiotensin 1
BÖtC: Bötzinger complex
CGRP: Calcitonin gene-related peptide
CIH: Chronic intermittent hypoxia
COX: Cyclooxygenase
CPAP: Continuous positive airway pressure
CTGF: Connective tissue growth factor
CVLM: Caudal ventrolateral medulla
CVO: Circumventricular organs
DOCA: Deoxycorticosterone acetate
eNOS: Endothelial nitric oxide synthase
ERSNA: Efferent renal sympathetic nerve activity
ESRD: End-stage renal disease
ET: Endothelin
H_2_S: Dihydrogen sulfide
HIF-1: Hypoxia inducible factor 1
HO-1: Heme oxygenase 1
HO-2: Heme oxygenase 2
ICAM-1: Intercellular adhesion molecule 1
IH: Intermittent hypoxia
IL-1β: Interleukin-1β
IL-6: Interleukin-6
MCP-1: Monocyte chemoattractant protein 1
MnPO: Median preoptic nucleus
MT: Metallothionein
NE: Norepinephrine
NF-κB: Nuclear factor kappa B
NK1: Neurokinin 1
NMDA: N-methyl-D-aspartate
nNOS: Neuronal nitric oxide synthase
NO: Nitric oxide
NOX: NADPH oxidase
NTS: Nucleus tractus solitaries
OSAS: Obstructive sleep apnoea syndrome
PAI-1: Plasminogen activator inhibitor 1
PGs: Prostaglandins
PVN: Paraventricular nucleus
RAAS: Renin-angiotensin-aldosterone system
ROS: Reactive oxygen species
RSNA: Renal sympathetic nerve activity
RTN: Retrotrapezoid nucleus
RTN/pFRG: Retrotrapezoid nucleus/parafacial respiratory group
RVLM: Rostral ventrolateral medulla
SFO: Subfornical organ
SHR: Spontaneously hypertensive rats
sLTF: Sensory long-term facilitation
SNS: Sympathetic nervous system
SOD: Superoxide dismutase
SP: Substance P
TGF-α: Transforming growth factor α
TGF-β1: Transforming growth factor β1
TNF-α: Tumor necrosis factor α
TRPV1: Transient receptor potential vanilloid 1
VCAM-1: Vascular cell adhesion protein 1
VEGF: Vascular endothelial growth factor
VRC: Ventral respiratory column.
